# Alzheimer’s Disease: A Molecular View of β-Amyloid Induced Morbific Events

**DOI:** 10.3390/biomedicines9091126

**Published:** 2021-09-01

**Authors:** Rajmohamed Mohamed Asik, Natarajan Suganthy, Mohamed Asik Aarifa, Arvind Kumar, Krisztián Szigeti, Domokos Mathe, Balázs Gulyás, Govindaraju Archunan, Parasuraman Padmanabhan

**Affiliations:** 1 Lee Kong Chian School of Medicine, Nanyang Technological University, Singapore 636921, Singapore; n1906466a@e.ntu.edu.sg (R.M.A.); balazs.gulyas@ntu.edu.sg (B.G.); 2 Cognitive Neuroimaging Centre, 59 Nanyang Drive, Nanyang Technological University, Singapore 636921, Singapore; 3 Department of Animal Science, Bharathidasan University, Tiruchirappalli 620024, Tamil Nadu, India; aarifam91@gmail.com; 4 Department of Nanoscience and Technology, Alagappa University, Karaikudi 630003, Tamil Nadu, India; suganthy.n@gmail.com; 5 Centre for Cellular and Molecular Biology, Hyderabad 500007, Telangana, India; akumar@ccmb.res.in; 6 Department of Biophysics and Radiation Biology, Semmelweis University, 1094 Budapest, Hungary; krisztian.szigeti@gmail.com (K.S.); domokos.mathe@hcemm.eu (D.M.); 7 CROmed Translational Research Centers, 1094 Budapest, Hungary; 8 In Vivo Imaging Advanced Core Facility, Hungarian Center of Excellence for Molecular Medicine (HCEMM), 1094 Budapest, Hungary; 9 Department of Clinical Neuroscience, Karolinska Institute, 17176 Stockholm, Sweden; 10 Marudupandiyar College, Thanjavur 613403, Tamil Nadu, India

**Keywords:** amyloid beta, Alzheimer’s disease, inflammation, gene regulation, organelle dysfunction

## Abstract

Amyloid-β (Aβ) is a dynamic peptide of Alzheimer’s disease (AD) which accelerates the disease progression. At the cell membrane and cell compartments, the amyloid precursor protein (APP) undergoes amyloidogenic cleavage by β- and γ-secretases and engenders the Aβ. In addition, externally produced Aβ gets inside the cells by receptors mediated internalization. An elevated amount of Aβ yields spontaneous aggregation which causes organelles impairment. Aβ stimulates the hyperphosphorylation of tau protein via acceleration by several kinases. Aβ travels to the mitochondria and interacts with its functional complexes, which impairs the mitochondrial function leading to the activation of apoptotic signaling cascade. Aβ disrupts the Ca^2+^ and protein homeostasis of the endoplasmic reticulum (ER) and Golgi complex (GC) that promotes the organelle stress and inhibits its stress recovery machinery such as unfolded protein response (UPR) and ER-associated degradation (ERAD). At lysosome, Aβ precedes autophagy dysfunction upon interacting with autophagy molecules. Interestingly, Aβ act as a transcription regulator as well as inhibits telomerase activity. Both Aβ and p-tau interaction with neuronal and glial receptors elevate the inflammatory molecules and persuade inflammation. Here, we have expounded the Aβ mediated events in the cells and its cosmopolitan role on neurodegeneration, and the current clinical status of anti-amyloid therapy.

## 1. Introduction

AD is a chronic debilitating neurological illness constituting 80% of dementia, primarily affecting the aging population above 65 years old. This devastating disorder is the one of the sixth leading causative of fatality worldwide, which has turned into a scourge of 21st century creating huge socioeconomic havoc globally and providing a burden to the caretakers [1]. AD is characterized by gradual neuronal degeneration that affects the cognitive function with severe memory impairment, lack of thinking, behavioral and social skills worsening activities of daily life. Epidemiological survey reveals that the global prevalence of AD increased in aging population where the incidence rate is higher in developed countries while in developing countries it is less than 1%. The World Health Organization (WHO) reported that globally around 47.5 million individuals are affected by AD as of 2018 with 7.7 million newcases every year, which is estimated to increase to 75.6 million by 2030 and 135.5 million by 2050, with expected cost of care at $1 trillion per annum [2,3]. Progressive changes in the brain of AD from asymptomatic to symptomatic transformation such as memory and behavioral disruptions are termed as AD continuum. AD is categorized as mild, moderate, and severe depending on the degree of symptoms that affects the day today activities [4,5,6]. In general, AD is classified into early onset AD (EOAD) and late onset AD (LOAD) based on the age prevalence. EOAD constitutes 5% of the total AD cases and is prevalent in people below the age of 65 years. EOAD is also termed as familial type AD (FAD) caused due to autosomal dominant mutation of genes coding for the APP, Presenilin 1 and 2 (PS1 and 2) located in Chromosomes 21, 14 and 1, respectively. People with Down’s syndrome (21st trisomy) have a higher risk for EOAD [7,8]. LOAD also termed as sporadic AD which frequently occurs in the elderly population above the age of 65 years, accounting 95% of total AD cases. Major genetic risk factor for LOAD is gene encoding for Apolipoprotein E (APOE) involved in cholesterol metabolism (Chromosome 19), which exists in three forms of APOE2, APOE3, APOE4. Among the alleles ApoE4 exhibited threefold increased risk for AD development [9]. In addition, other factors such as aging, diet, lifestyle, environmental factors, and chronic metabolic disorders such as type II diabetes mellitus, hypertension and vascular disorders intensify the pathogenesis of LOAD [10]. Pathological trademarks of AD involve classical positive lesion comprising of amyloid plaques composed of Aβ peptides in the synaptic terminal of brain parenchyma and in the cerebral blood vessels leading to congophilic angiopathy/cerebral amyloid angiopathy (CAA); NFTs composed of paired helical filaments with hyperphosphorylated tau in the axonal region, neuropil threads, and dystrophic neurites accompanied by microgliosis and astrogliosis [11]. In addition, pathological features such as neuronal loss and synaptic dysfunction representing the core negative features of AD were also observed along with plaques in the cortical mantle and tangles in limbic and association cortices. Neuropathology of AD is associated with neuronal loss and atrophy in the temporofrontal cortex inducing further deposition of amyloid plaques and tangled bundles of fibers leading to enhanced migration of monocytes and macrophages in cerebral cortex provoking neuroinflammation [12,13]. Aβ peptide is the key factor in AD pathogenesis and fibrogenesis of Aβ peptide, triggers a cascade of events such as hyperphosphorylation of tau and NFT formation, ER stress, disruption in Ca^2+^ homeostasis, mitochondrial dysfunction, microgliosis and astrogliosis inducing neuroinflammation eventually leading to neuronal death [14]. Therapeutic intervention approved by FDA for AD includes cholinesterase inhibitors (Donepezil, Galantamine, Rivastigmine and Tacrine) and N-methyl-D-aspartate receptor (NMDAR) agonist (memantine) which are effective only for mild to moderate dementia and antipsychotic drugs for treatment of behavioral disturbances [15,16]. These therapeutic interventions possess severe side effects and have limited therapeutic efficacy on cognitive function, as these drugs only relieve the symptoms with no effect on progression of disease. Currently researchers are focusing on development of disease modifying drugs, which can slow or reverse the progression of disease [17]. However, most of these drug molecules failed in the clinical trials due to the mystery in understanding AD pathogenesis. Hence, the present review focuses on unravelling the molecular mechanism and biochemical pathways leading to pathogenesis of AD, which might help researchers in reassessing AD pathogenesis in different perception, thereby providing novel ideas for identification of therapeutic strategies to combat AD.

## 2. APP Processing

### 2.1. Post-Translational Modification of APP Alters Aβ Production

**Figure 1:**
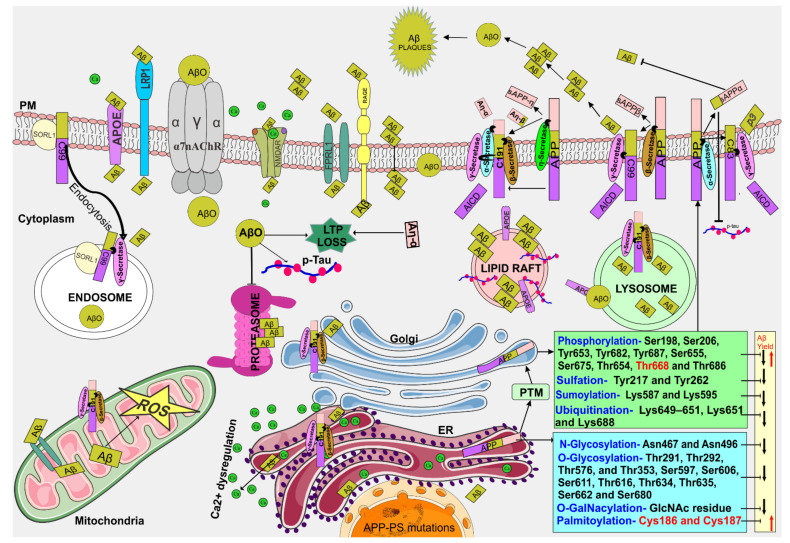
APP processing and Aβ generation: The PTM of APP alters Aβ production. The APP processing by the secretases (α, γ, β and η) at plasma membrane (PM), mitochondria, ER, lysosome, GC and lipid rafts generate its metabolites notably Aβ. Aβ undergoes oligomerization and plague formation at extra cellular matrix (ECM) and the ECM Aβ enters the cytoplasm through direct PM passing and receptor mediated internalization. Intra cellular Aβ accumulation in the cell organelle impairs its physiological functions.

APP, a type I integral membrane protein is found in both mammalian and non-mammalian cells. Three members of APP are present in mammals which are APP, the APP-like protein-1, and 2 (APLP1 and APLP2) [18]. In humans, APP is encoded in the chromosome arm 21 (21q21.3) with approximately 240 kb size which has 18 exons [19]. The promotor sequence of APP does not have TATA or CAAT boxes but has a sus sequence where the transcription factors such as SP-1, AP-1, and AP-4 binds to the promotor site and commences the gene expression [20]. SP1 is a zinc finger protein that binds to the GC rich region of APP promoter, which facilitates the binding of RNA polymerase II. Recent reports reveal that binding of lead (Pb^2+^) on the Zn^2+^ site of zinc finger 3 of SP1 (SP1-f3) protein promotes the APP overexpression and it could cause a high chance of amyloidogenesis [21]. The alternative splicing of APP mRNA engenders several isoforms, which vary from 365 to 770 amino acid residues. The Aβ1-42 is embedded in the proteins such as APP695, APP751, and APP770. The APP mRNA is localized in multiple tissues of the body. However, the APP695 is predominantly present in neuronal cells and the other two isoforms are expressed in other tissues [22,23]. Pre-mature APP protein undergoes several post-translational modifications (PTM) including N- and O- glycosylation, sumoylation, phosphorylation, ubiquitination, sulfation, and palmitoylation (Figure 1) [24,25,26]. N- and O- glycosylation occurs inside the ER, the oligosaccharyltransferases catalyses the N- glycosylation in Asn467 and Asn496 [27]. O- glycosylation is identified in several sites of APP770 including Thr291, Thr292, Thr576, and Thr353 [28], and Ser597, Ser606, Ser611, Thr616, Thr634, Thr635, Ser662 and Ser680 [24]. Furthermore, the single β-N-acetylglucosamine (GlcNAc) residue is added in serine or threonine residue that leads to the formation of O-GalNAcylation, which plays an important role in non-amyloidogenic processing of APP [29]. In GC, APP undergoes phosphorylation in 10 residues of both ecto- and cytoplasmic domains, which are Ser198, Ser206, Tyr653, Tyr682, Tyr687, Ser655, Ser675, Thr654, Thr668 and Thr686 [25,30]. The cytoplasmic Ser655 and Thr654 are phosphorylated by Ca^2+^/calmodulin-dependent protein kinase II (CaMKII) [31]. A decreased level of Ser655 phosphorylation and enhanced level of Thr668 phosphorylation stimulates Aβ generation [25,32]. Tyr682 and Tyr687 phosphorylation have been found in the AD brain but not in a healthy brain, as well as in APP overexpressed cells [33]. About 10% APP undergoes palmitoylation process in the ER by the DHHC-7 and DHHC-21 (palmitoyl acyltransferases) at the site of Cys186 and Cys187. Palmitoylated APP enriched in the lipid rafts where the Beta-Site APP Cleaving Enzyme 1 (BACE-1) level is also higher facilitating the Aβ production. Thus, suppressing the APP palmitoylation has therapeutic competence against Aβ through targeting both α- and β-secretase cleavage [26]. The Lys649, Lys 650, Lys651 and Lys688 are the ubiquitination sites of APP where the attachment of ubiquitin associates in the protein degradation, interaction, and the trafficking process. In addition, the sumoylation of APP at Lys587 and Lys595 is catalysed by the enzyme small ubiquitin-like modifier 1 and 2 (SUMO-1 and SUMO-2). Both the ubiquitination and sumoylation process decreases the Aβ level for example high-level of APP sumoylation reduce the Aβ production [34,35,36]. The sulfation occurs at Tyr217 and Tyr262 residues of APP, however the exact sulfation sites and functions are not completely discovered [37]. On the other hand, the PTMs analysis of AD molecules revealed citrullination (Arg301), phosphorylation (Ser366, Ser441), and methylation (Lys624, Lys699) of APP protein [38]. In addition, citrullination (Arg5→Cit) and deamidation (Asn27→Asp) of Aβ fragments affects the fibrillation rate of Aβ [39]. The physiological function of APP is still unclear however the overexpression of wild-type APP promotes the cell proliferation, neurotoxic and neurotrophic protective effects [40]. Overall, the PTM of APP venues, its bi-directional therapeutics opportunity via enhancing ubiquitination, O-GalNAcylation, simulation and diminishing phosphorylation and palmitoylation.

### 2.2. APP-Secretases Processing and Aβ Generation

APP is processed by membrane proteases such as α-, β-, γ- and η-secretases. Cleavage of α- or β-secretases, followed by γ- secretase leads to generation of non-pathological (by α-secretase) or pathological (by β-secretase) fragments (Figure 1) [41]. α-secretase is a type of metalloprotease and disintegrin (ADAM) family member. Further, ADAM 9, 10 and 17 has α-secretase activity. ADAM 10 is the primary secretase that cleaves the APP in neurons. α-secretase resides within the Aβ domain of APP which cleaves and produces the extracellular soluble APPα (sAPPα) and C-terminal fragment (CTF)-α-83 or C83 [42]. BACE-1 is a type-I transmembrane aspartyl protease which cleaves APP at Asp1 or Glu11 sites generating the soluble APPβ (sAPPβ), the CTFβ-99 (C99) or CTFβ-89 (C89) [43]. A673V mutation in APP exhibits shifts BACE-1 cleavage from Glu11 to Asp1 site, increasing the level of C99 and C99/C89 ratio [44]. θ-secretase or BACE-2 is homologous to the BACE-1, which cleaves APP at Phe19 of the Aβ domain and produces CTF-80. However, BACE-2 transgenic mice did not show Aβ overproduction and cognitive deficits [45,46]. γ- secretase is a complex protein comprising of PS1 and PS2, nicastrin, anterior pharynx-defective 1 (APH-1) and presenilin enhancer (PEN-2) subunits. PS1 and PS2 are the catalytic unit and nicastrin, APH-1 and PEN-2 are the regulatory units that mainly functions in substrate recognition [47,48]. Several reports reveal that the γ- secretase activating protein (GSAP) regulates the γ- secretase specificity, which induce conformational change of PS1 and substrate recognition. GSAP significantly and selectively elevates the Aβ production without altering γ- secretase normal functions such as notch cleavage [49,50,51]. γ- secretase cleaves the C83 and C99 resulting in the production of fragments such as P3, varied length of Aβ(38-49), and APP intracellular domain (AICD) [42]. C89 processing by γ- secretase reveals generation of truncated Aβ11-40/42 (Aβ’) peptides [52]. In addition, the Aβ from the AD brain exhibits prominent modifications in N- terminal site of Aβ including truncation (Aβ’), glutamate conversion to pyroglutamate and isomerization of L-Asp to D-Asp, which results in the loss of N- terminal charge that turns the peptide more hydrophobic [53]. γ- secretase assay with recombinant APP-CTFs in human induced pluripotent stem cell (iPSC) or cell-free system showed elevated level of 42:40 ratio higher in Aβ′ > Aβ > P3 [54]. η-secretase is a membrane-bound matrix-metalloproteinase such as MT5-MMP, which cleaves APP at 504/505 residue and generates truncated sAPP-η and CTF-η (C191). ADAM-10, BACE-1 and γ- secretase involved in CTF-η cleavage produces Aη-α and Aη-β peptides of ECM, ACID into the cytoplasm. Hippocampal long-term potentiation (LTP) is lowered upon the treatment of synthetic Aη-α in mice hippocampal slices [55]. About 440 mutations in APP, PS1, and PS2 were identified to be linked to FAD (https://www.alzforum.org/mutations searched on 28 August 2021), and several pathogenic research models were developed thereafter. PS mutations decreased γ-secretase sensitivity, which increases the number of cuts in a single substrate. γ- secretase cleavage in multiple sites of C99 (γ, ς and ε-sites) generates intermediate products 43, 45, 46, 48, 49 and 51 amino acids, which were further cleaved producing final product Aβ40/Aβ42. FAD APP mutations become partially resistant to the γ- secretase cleavage. The N-terminal APP mutations show subtle effect on γ- secretase cleavage efficiency and Aβ40/42 specificity. However, the C-terminal APP mutations show skewed cleavage and aggregation prone Aβ42 specificity [56]. Several Aβ degrading enzymes (ADEs) were reported which catalysts the proteolytic degradation [57]. Currently, researchers are focusing on the disease-modifying anti-amyloid therapy targets to alter Aβ production/aggregation which includes ADEs, BACE-1, PS1, and GSAP (detailed [49,57]).

### 2.3. Intra-Cellular Aβ

Aβ is observed as an extracellular product of APP, but later the incidences of intracellular Aβ production were reported. APP is found in several compartments such as ER, trans-Golgi network (TGN), mitochondrial membranes, endosomes, and lysosomes (Figure 1) [58,59,60]. In particular, the exosomes play a significant role in transporting the APP and APP-CTF where the Aβ release could occur wherever the β- and γ- secretases are co-localized within the exosomes. AD patient’s extracellular vehicles (EVs) or exosomes has increased levels of cytotoxic Aβ and prion protein (PrP) which get transferred to neighbor neurons that could serve as a diagnostic and in therapeutic applications [61,62,63]. The intracellular Aβ liberation is found in cells with APP_Swe_ but not in the cells with wild type APP [64]. Sortilin-related receptor 1 (SORL1) plays a critical role in late-onset AD, where it recovers the uncut APP from the PM through internalization into endosomes [65]. Endosomes are acidic in nature where the BACE-1 actively interacts with APP and generates Aβ42 [66]. The decreasing intracellular Aβ lead to extracellular plaque formation, and this is evident in Down syndrome patients [67]. In addition, the reuptake of the extracellular Aβ is found in the cells through the Aβ interaction with several biomolecules including lipids, proteins, and proteoglycans. Aβ can instantly aggregate to form lower molecular weight dimers to fibrils that lead to amyloid plaques in AD brain. In Aβ aggregation, the intermolecular hydrogen bonds of β-strand of Aβ peptides form the cross-β structural pattern, and the hydrogen-bonded, parallel β-sheeted Aβ peptides which induce fibril formation in the presence of tissue transglutaminase (tTg). These Aβ aggregates exhibit toxic effects mostly from dimers itself, which interrupts learning and memory and LTP [68,69]. It was anticipated that the elevated amount of Aβ oligomers (AβOs) may be non-specifically conducted through the direct interaction with negatively charged phospholipid bilayers [70]. Receptor-mediated Aβ internalization was found in α7 nicotinic acetylcholine receptor (α7nAChR) [71], APOE [72], formyl peptide receptor-like 1 (FPRL1) [73], NMDA [74] and receptor for advanced glycation end products (RAGE) [75] receptors. Aβ oligomerization is found intracellularly in multiple sites of the cell such as plasma, endosomal and lysosomal membranes, and lipid rafts [76,77]. The Tg2576 AD model exhibit Aβ dimers which predominantly accumulate in the lipid rafts along with APOE and p-tau indicating the fact that lipid rafts are the important interaction site for the above proteins [78]. Recent reports reveal that AβO directly inhibits the ubiquitin-proteasome system, which is shown in both animals and cell lines. The proteasome inhibition upon Aβ interaction leads to accumulation of tau protein. On the other hand, Aβ internalization is also detected in the mitochondria, which interferes in its function by diminishing the ETC III and IV and reduced oxygen consumption [79]. Christian and his colleagues reported that Aβ42 treatment in mouse neuronal primary cells and hippocampal slices induced tau phosphorylation (p-tau), while sAPPα treatment decreased the levels of Aβ42 and p-tau proteins [80]. In addition, the BACE-1 and GSK3β activities were also observed to be dropped in cell culture and APP-PS animal models, associated with decline in tau hyperphosphorylation upon treatment with sAPPα [81]. sAPPα activates mitogen-activated protein kinase (MAPK)/extracellular signal-regulated kinase (ERK) which results in neurotrophic and neuroprotective activity [82]. The LTP of transgenic mice is recovered by sAPPα through microglial invasion on the sites of Aβ deposits, which upregulates insulin-degrading enzymes (IDE) leading to Aβ clearance and restoration of spatial learning and synaptic plasticity [83].

## 3. Tau Pathology

Major neuropathological hall mark of AD in addition to senile plaque is the presence of intra neuronal neurofibrillary tangles composed of hyperphosphorylated tau protein. Tau protein is a microtubule-associated phosphoprotein (MAP) abundant in axons of the neurons engaged in boosting the tubulin assembly into microtubules and its stabilization in the brain [84]. Microtubule associated protein tau gene is encoded by Chromosome 17. Tau exists in six isoforms (352–445 amino acids) formed by alternate splicing of exon 2, 3 and 10, which vary by the presence or absence of 29 or 58 amino acids insert in the N-terminal part and by three or four microtubule binding repeats (3R or 4R) in the C-terminal end. Exon 10 on alternative splicing forms two isoforms, four repeats isoform tau (4R-tau) or three repeats isoform tau (3R-tau), which play a vital role in microtubule binding [85]. In addition, examination of healthy adult brain regions shows N-terminal fragments of tau ranging from 40 kDa and 45 kDa and C-terminal fragments of tau ranging from 17 kDa to 25 kDa in the age group between 18 and 108 years where the truncations of tau at the Asp421 and Glu391 residues are believed to be an aggregation promoting factor of AD [86]. Tau protein consists of hydrophilic acidic residue rich N-terminal domain, flanked by basic proline rich domain, microtubule binding domain (MBD) and downstream is the C-terminal domain. In the adult brain, the tau protein consists of two to three moles of phosphate per mole of the protein, with a rich source of proline-glycine motifs facilitating the folding of tau protein [87]. Post translational modification of tau primarily serine/theronine directed phosphorylation and glycosylation modulates the affinity of tau for microtubules. Phosphorylation of tau, which starts during brain embryonic development of the fetal brain is comparatively higher when compared to adult brain [88]. In addition, deamidation (Asn279→Asp279) of 4R-tau involving degeneration of several brain regions acts as a biomarker for AD where the deamidated sequence specific antibodies helps to detect the marker protein [89]. A major role of the tau protein is to facilitate assembly of the microtubules and maintain its structure for normal axoplasmic flow. Assembly of microtubules is dependent on the extent of phosphorylation, as hyperphosphorylation affects the assembly and stability of microtubules. In addition to its interaction with tubulin, tau also binds with the SH3 domain of Src family tyrosine kinases revealing its role in cell signalling [90]. Tau protein plays a putative role in maintaining the stability of chromosomes [91].

### 3.1. Aβ Peptide Promotes Hyperphosphorylation of Tau Protein

**Figure 2:**
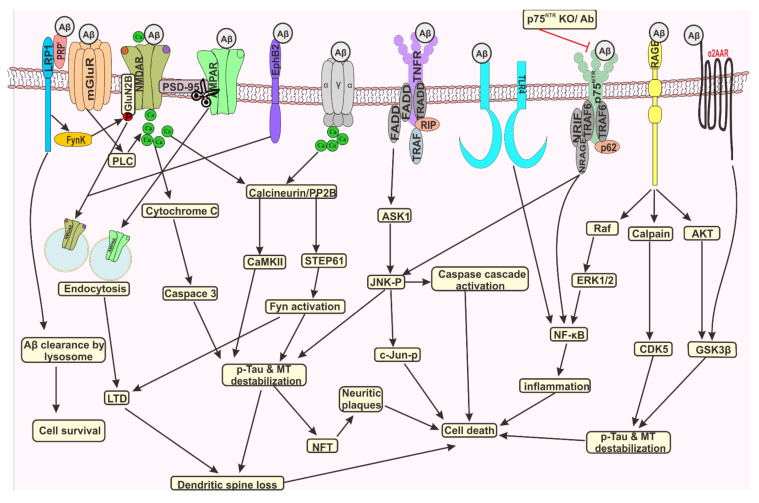
Signaling of Aβ in memory impairment, tau hyperphosphorylation and cell death in AD: LRP1 receptor facilitates the Aβ clearances as well activates the FynK. Aβ binding with receptors such as mGluR, NMDAR, AMPAR, EphB2 triggers the LTD via NMDAR and AMPAR surface removal. In other hand, prolonged activation of NMDAR elevates the intracellular Ca^2+^ level which increases caspase 3. The inflammation is induced by the activation of NF-κB upon Aβ mediated activation of TLR-4, p75NTR and RAGE receptors. The tau hyperphosphorylation is induced by several kinases including GSK3β, CDK5, JNK, FynK upon Aβ binding with most of the receptors which destabilizes microtubules, forms the NFT and neurotic plaques. The LTD, NFT, inflammation, increased caspase cascade leads to dendritic spine loss and cell death.

Tau pathology in AD is mainly contributed by the oxidative stress and inflammatory response induced by Aβ toxicity. Several kinases are engaged in the phosphorylation of tau protein (p-tau) which includes proline dependent kinases such as glycogen synthase kinase-3 (GSK3), dual specificity tyrosine-phosphorylation-regulated kinase 1A/B (Dyrk1A/B), cyclin-dependent protein kinase-5 (CDK5), and mitogen activated protein kinases (MAPK) (p38, Erk1/2, and JNK1/2/3); proline independent kinase such as tau-tubulin kinase 1/2 (casein kinase 1α/1δ/1ε/2), microtubule affinity regulating kinases, phosphorylase kinase, cAMP-dependent protein kinase A, C and N, CaMKII and tyrosine protein kinases such as Src family kinase (SFK) members (Src, Lck, Syk, and Fyn) and Abelson family kinase members, ABL1 and ABL2 (ARG) which were considered as target sites for AD therapeutics (Figure 2) [92]. Among the tau specific kinases, GSK3β is the key enzyme in hyperphosphorylation of tau protein, which phosphorylates at least 15 residues of Tau protein. In AD, soluble and fibrillar Aβ peptides tend to be more toxic rather than amyloid plaques. Several pieces of evidence revealed the inter-relationship between Aβ toxicity and tau pathology, which remains still unclear. One hypothesis proposed that soluble Aβ binding to α7nAChR irreversibly activates tau specific kinases promoting persistent p-tau protein at three proline directed sites that enhances tau protein dislocation from axon to dendrites and synaptic junction, disrupting axon/dendritic transport followed by neurofibrillary lesion, dendritic breakdown leading to neurofibrillary tangles formation [93]. Evidence also revealed that enhanced intracellular Ca^2+^ due to Aβ induced mitochondrial dysfunction and ER stress activates caplapin dependent cleavage of P35 and P39 generating P25 and P29 fragments, which in turn activates Cdk5 followed by activation of GSK3β leading to hyperphosphorylation of tau protein forming NFT in AD brain as observed in transgenic animals [94,95]. The enhanced tau phosphorylation is associated with difficulty in spatial learning in transgenic mice expressing increased GSK3β [96]. King et al., stated that oxidative stress induces nitration of tyrosine residue in tau protein and disulphide bridge formation between tau protein facilitates abnormal folding and detachment of tau from microtubules thereby, promoting aggregation [97]. Transient activation of PKA in AD induces continuous hyperphosphorylation of tau in both PKA and non-PKA sites making tau protein more susceptible to successive phosphorylation by GSK3β, revealing the fact that stimulation of PKA plays a crucial part in the commencement of the AD [98]. Another enzyme, MAP affinity regulating kinase (MARK) also phosphorylates the KxGs motif present in MBD of tau protein. Although upstream regulation of MARK is not clear, a recent study revealed that GSK3β activates MARK2 inducing phosphorylation of Ser-262 of tau [99]. In addition, ERK2 activation in neurons also promotes p-tau reducing its ability to stabilize microtubules [100]. The rate of p-tau depends on the initial phosphorylation site Ser 396 and Ser 235, following the phosphorylation of predecessor Ser 400 [101], and prime phosphorylation of Thr 231 [102], which has significant role in modulating the function of tau protein, i.e., stabilizing the microtubules. In addition to tau phosphorylation, these kinases are also involved in APP processing also, e.g., GSK3, especially GSK3α, promotes Aβ formation from APP offering new approach, that inhibition of GSK3α might attenuate amyloid plaques and NFT formation [103]. CDK5-p25 regulates APP processing leading to Aβ production by phosphorylating Thr 668 residue of APP. Other factors such as stress in the ER and life stress also influence p-tau [104]. A recent study reported that increased expression of P75 neurotrophin receptor (p75NTR) the pan-receptor for Aβ peptide promoted Aβ neurotoxicity by inducing the production of Aβ via endocytosis of APP and BACE-1, phosphorylation of tau protein via calpain/CDK5 and AKT/GSK3β pathways. Calpain promotes truncation and activation of GSK3β. The CDK5 activator protein p25 preferentially binds with and activates GSK3β [105]. In vitro studies revealed a cross talk between the calpain/CDK5 and AKT/GSK3β pathways downstream of Aβ/p75NTR signalling in the regulation of p-tau levels in AD (Figure 2) [106].

Three major phosphatases PP1, PP2A, PP2B and PP2C play a major role in de-phosphorylation of tau protein among, which PP2A has predominant role whose activity is reduced by 30% in the AD brain [107]. As an inhibitor of PP2A, Aβ deposition and estrogen deficiency causes phosphorylation of Y307 subunit of PP2A inactivating the protein ability to dephosphorylate hyperphosphorylated tau protein, which in turn leads to the NFT formation [108]. Another report revealed that abnormal increase in mitochondrial ROS level in Aβ inhibits PP2A and PP5 activating JNK and Erk1/2 pathways leading to apoptosis of neuronal cells [109]. Bolmont et al. reported that when tau mutant transgenic mice were intracerebroventricularly administrated with APP transgenic mice brain extract NFT formation was observed, depicting the fact that Aβ acts as upstream factor of tau pathology [110]. Another report revealed that Aβ induces upregulation of gene coding for tyrosine kinase 1A (DYRK1A) promoting hyper-phosphorylation of tau protein, causing microtubule disassembly. Similarly, the cytotoxicity of Aβ oligomers is promoted by the tau protein, revealing the fact that Aβ triggers tau pathology, while tau protein intercede toxicity of the Aβ protein and both acts synergistically, enhancing AD pathology [111,112]. Despite these studies, the connection between the GSK3β and PP2A with Aβ induced oxidative stress is still elusive. The p-tau and aggregation are also influenced by PTM of tau including glycosylation, nitration, truncation, acetylation, sumoylation, ubiquitination, and polyamination.

### 3.2. Molecular Mechanism of p-Tau Mediated Neurodegeneration

In AD brain cytosol, 40% of abnormally hyperphosphorylated tau exists in oligomeric and nonfilamentous form. Hyperphosphorylated tau forms of PHF in the soma and thread-like lesion termed as neurophil neuritis found in the grey or white matter and senile plaque are associated with dystrophic neuritis. The concentration of total tau and p-tau in CSF determines the stage of AD [113]. Hyperphosphorylated tau not only bind with normal tau protein promoting self-assembly into PHF, but it also binds with microtubule associated proteins (MAP1 and MAP2) and actin disrupting the self-assembled microtubules leading to zeiosis of cell membrane ultimately causing neurofibrillary degeneration (Figure 2) [114]. Hyperphosphorylated tau is released within the vesicles formed by pinching of destabilized membrane, which are taken up by the neighboring cells by a process called endocytosis, thereby sequestering the healthy tau protein to hyperphosphorylated form via its prion like nature spreading from one neuronal cell to other disrupting its cytoskeletal structure, organelle destabilization, interrupting protein synthesis ultimately leading to induction of zeiosis [115]. Extracellular soluble tau is taken up by the healthy neurons through muscarinic receptor, which play significant role in the neuronal signal transduction [116]. Based on these facts, Morozova et al., reported that uptake of hyperphosphorylated tau via muscarinic receptor, in addition to endocytosis, also translocates into the nucleus, alters the protein expression, move to synapse, and impairs the mitochondrial function [117]. As the level of phosphorylated tau increases in neurons with disease progression, it disrupts the microtubule stability and damages the cytoskeletal components triggering neurodegeneration [118]. Pathways involved in removal of damaged, misfolded, and aggregated protein are the ubiquitin-proteasome and autophagy lysosomal pathways. The brains of AD patients were characterized by accumulation of polyubiquitylated tau proteins with reduced proteasome activity. The proteasome activity reflects the amount of PHF in AD brain and its activity is inhibited by the hyperphosphorylation of tau protein illustrating the fact that hyperphosphorylation and proteasome inhibition are inter-related [119].

## 4. Aβ in Mitochondria Dysfunction

The central nervous system has high metabolic demand which is met by the abundant mitochondria present in the neurons. Mitochondria regulate the life and death cycle through various cellular regulatory process such as ATP production, maintaining intracellular Ca^2+^ homeostasis, reactive oxygen species production, detoxification and apoptosis [120]. Mitochondria generates ROS such as superoxide (O_2_^−^), hydroxyl radical (OH), and hydrogen peroxide (H_2_O_2_) which are counterbalanced by the antioxidants such as super oxide dismutase, catalase, glutathione peroxidase (GPX), glutathione reductase (GR) and sirtuins (mammalian class III histone deacetylases) [121,122]. Imbalance in the proxidant and antioxidant level leads to protein oxidation, lipid peroxidation and DNA damage altering the mitochondrial membrane potential, disturbing Ca^2+^ homeostasis, enhancing cytochrome C ultimately leading to apoptosis. Sirtuins (Sirt) in the mitochondria of neurons regulates the transcription and antioxidant enzymes (SOD2 and catalases) activities. Sirt1 regulates the peroxisome proliferator-activated receptor-gamma coactivator1 alpha (PGC-1α), APP metabolism and Aβ level [123]. Sirt3 play an important role in 8-oxoguanidine DNA glycosylase 1 (OGG1) stabilization and deacetylation, promoting mtDNA repair via nuclear enzyme. Aβ-induced reduction in Sirt1 and Sirt3, upregulates the tau level and acetylation [124,125]. The dynamin-related protein 1 (Drp1), mitofusins1 and 2 (Mfn1 and Mfn2) and optical atrophy 1 (Opa1) are the key fission/fusion proteins that regulates the mitochondrial dynamics via maintaining the assembly and stability of ECT super-complex structure, altering mitochondrial structure and distribution of mitochondria throughout the neurons [126].

### Mitochondrial Dysfunction by Aβ

Mitochondrial dysfunction was found in the post-mortem brain samples of AD patients and their platelets, AD transgenic mice and in-vitro over expressed mutant APP or external Aβ treatment [127,128,129,130]. Post-mortem brain samples of the AD patients exhibit an elevated level of oxidative damage, increased mtDNA and cytochrome oxidase in neuronal cytoplasm, which indicates accumulation of mitochondrial products upon degradation [128]. The glucose metabolism of the AD patient’s brain is down-regulated, and the late-onset AD brain showed reduced mitochondrial membrane potential through metabolic shift from mitochondrial oxidative system to glycolysis [129,130]. EOAD patient’s mRNA investigation revealed down-regulation of complex I and upregulation of complexes III and IV of ETC when compared to normal subjects indicating the highest demand in energy production [131]. AD patient platelets isolated and fused with human neuroblastoma (SH-SY5Y) and teratocarcinoma (NT2) cells showed reduced endogenous mtDNA. These cells showed elevated Aβ production along with mitochondrial dysfunction with lower cytochrome oxidase activity, higher free radical generation, reduced mitochondrial membrane potential and altered calcium homeostasis [127]. The transgenic mice models such as APP_swe/Lon_ and double transgenic APP/PS1 showed reduction in the mitochondrial chaperon hsp70, reduced glucose metabolism, impaired Cu/Zn SOD activity, decreased mitochondrial membrane potential and ATP level, increased mitochondrial permeability transition, a decline in respiratory function, and increased mitochondrial oxidative stress [132,133,134]. Overexpression of APP_Swe_ on PC12 cells depicts increased level of oxidative stress and mitochondrial dysfunction mediated by various caspases and the stress-activated protein kinase pathway, elevated level of ROS, reduced ATP generation [135,136]. In addition, APP_Swe_ MC17 cells exhibited elevated level of Aβ production, which causes the imbalance in the mitochondrial fission/fusion protein where the dynamin-like protein 1 (DLP1) and OPA1 levels are decreased and the Fis1 level is enhanced. These changes results in mitochondrial fragmentation which promotes mitochondrial and neuronal dysfunction [137]. Expression of transcription genes (anti-oxidative and mitochondria-related proteins) in AβO treated BV2 (glial) and SH-SY5Y (neuronal) cells, transgenic mice (Tg-AD), and human AD brain revealed enhanced expression of Sod2, Dnm1l, Bcl2 genes and reduction of Gpx4, Sirt1, Sirt3, mt-Nd1, Sdha and Mfn2 genes. In addition, reduction in cell viability was observed with enhanced ROS production and impaired MMP Tg-AD mice showed significant down-regulation of Sirt1, Mfn1 and mt-Nd1 and upregulation of Dnm1l. In addition, the human AD brain showed alteration in microRNA pattern, which is responsible for the reduced Sirt1 expression [138]. SIRT1 activates the transcription of PGC-1α, which improves the antioxidant capacity inducing the expression of SOD and GPX in cells. Reduction in PGC-1α causes impairment in mitochondrial biogenesis, the one-month aged AD mice showed no significant AβO deposition with elevated PGC-1α, whereas the six months old AD mice exhibited the high level of AβO deposition with significant reduction in PGC-1α [139].

APP has a binding motif for TOM40 that impairs the routine function of mitochondria, the APP binding with TOM40 impedes the COX (IV and Vb) transportation, which results in diminished COX activity and increased ROS production [140]. Mitochondria-associated ER membrane is a communication point between the ER and mitochondria.

**Figure 3:**
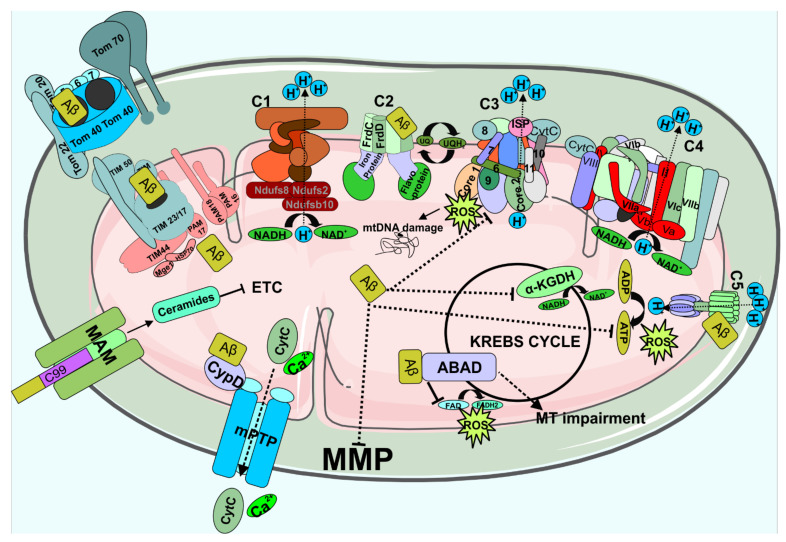
Mechanism of Aβ induced mitochondrial dysfunction: Aβ enters the mitochondria (MT) through the TOM40 and TIM 23 complex. Aβ interacts with the Aβ-binding alcohol dehydrogenase (ABAD), Cyclophilin D and electron transport complexes (ETC) (C2, C3 and C5) which enhance the ROS production, Cytochrome C liberation, ROS mediated MT-DNA damage, MT impairment and mPTP opening where CytC exported to the cytoplasm. In addition, MAM bound APP fragment (C99) activates ceramides that inhibits the ETC.

Mitochondria-associated membrane (MAM) play a crucial role in calcium transport, phospholipids synthesis and mitochondrial fission–fusion dynamics. Remarkably, MAM has enhanced the level of C99 and γ- secretase where the Aβ is generated via amyloidogenic pathway [58,141]. In addition, the C99 accumulation at MAM activates sphingomyelinase that generates ceramides, which leads to inhibition of mitochondrial respiration and apoptosis [142,143]. Further, the Aβ co-localization is found with the complex II of the mitochondrial ETC, which indicates that the Aβ pass the outer and inner membranes of the mitochondria [144]. Hansson Petersen et al. studied the import mechanism of Aβ into the mitochondria using immunohistochemistry, immunoblotting, immunoelectron microscopy and flow cytometry techniques [145]. Decline in Aβ transportation was observed in mitochondria exposed to antibodies towards mitochondrial receptors Tom20 or Tom70, or the general mitochondrial import pore of the outer membrane Tom40, indicating the fact that Aβ gets into mitochondria via the TOM40 complex, which translocate Aβ into the inner membrane via TIM22 complex. Aβ interacts with different mitochondrial proteins upon entry into the matrix including amyloid binding alcohol dehydrogenase (ABAD), Complex V and cyclophilin D. Complex V of ETC produces ATP, when Aβ interacts with Complex V α-subunit disturbing its energy production (Figure 3). Alcohol dehydrogenase catalyses the reduction of nicotinamide adenine dinucleotide (NAD) to NADH. ABAD has a direct link with the Aβ to mitochondrial toxicity, the crystal structure of Aβ bound ABAD shows the deformation of active site that affects the NAD binding. ABAD over expressed mice in Aβ enriched environment reveal elevated oxidative stress and impaired memory [146]. Complex V is regulated by the addition of O-linked N-acetylglucosamine (O-GlcNAcylation) which is catalysed by O-GlcNAc transferase, Aβ interrupt the binding between ATP5A and O-GlcNAc transferase [147]. The mitochondrial permeability transition pore (mPTP) regulates the apoptotic pathway via Ca^2+^ and apoptotic signalling molecules from the matrix [148]. The Ca^2+^ enters the mitochondria through either mitochondrial membrane Ca^2+^ uniporter (MCU) or voltage-dependent anion channel (VDAC) which results in unlocking the mPTP and generates the ROS [149]. The mPTP is regulated by the Cyclophilin D, the interaction of Aβ with Cyclophilin D inhibits Cyclophilin D leaving the mPTP pore open [150]. Further, the specific loss of oligomycin sensitivity conferring protein (OSCP), a subunit of Complex V upon interaction with Aβ results in mPTP activation, decreased ATP production, and increased oxidative stress (Figure 3) [151]. The oligomeric Aβ treatment showed an entry of extracellular calcium into the mitochondria, which causes the mitochondrial mediated apoptosis via opening the mPTP channels and releasing the cytochrome C [152]. Cym1/PreP proteasome degrades the pre-sequence of the protein and Aβ peptides. In oxidizing condition, the Cys527 and Cys90 can form a disulphide bridge that results in PreP inactivation elevating the amount of Aβ in mitochondria. Aβ inhibits the degradation of pre-sequence peptides, which results in the dysfunction of preprotein processing leading to imbalanced organellar proteome and multiple mitochondrial defects including reduced membrane potential, oxygen conception and increased ROS [153,154]. Aβ exposed brain vascular endothelial cells show increased level of inter mitochondrial calcium (Ca^2+^) and reduced cytosolic Ca^2+^ resulting in enhanced amount of oxygen conception, higher ATP production and increased ROS generation, which in turn contribute to cerebrovascular dysfunction [155].

## 5. Aβ in ER Stress

The ER is a functional organelle that coordinates the proteostasis of the eukaryotic cells including protein biosynthesis, folding, assembly, trafficking, and ruining. Accurate protein folding according to intracellular or extracellular signal is crucial for normal cell survival and physiological functions [156]. The ER maintains the Ca^2+^ homeostasis, where the lumen of the ER has the utmost level of Ca^2+^ ions within the cell via the active transportation of Ca^2+^ through Ca^2+^ ATPase channel [157]. Ca^2+^ plays a vital in the processing and folding of new-born proteins, since these mechanisms are strictly Ca^2+^ dependent so high Ca^2+^ level is required for the proper functioning [158]. Any disruption in ER function such as dysregulation of Ca^2+^ homeostasis, inhibition of PTM and hypoxia heaps the accumulation of unfolded or misfolded proteins, which leads to the ER stress due to the huge unwanted protein load and the long-term accumulation of these proteins induce the cell damage [159]. To decrease the unfolded protein level, the cells activate several cellular systems including UPR and ERAD, which protects the cells against the toxic proteins that augments the ER capacity and quality control [160].

### ER Dysfunction by Aβ

**Figure 4:**
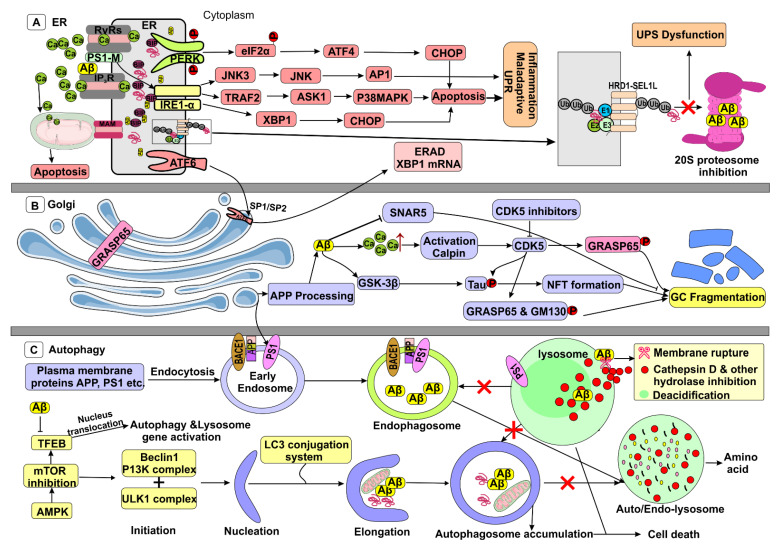
The molecular mechanisms of Aβ prompted ER, GC and Lysosome dysfunction: Aβ induces the ER stress via increasing Ca^2+^ outflux from ER through IP_3_R and RyR which accelerates the apoptosis via mitochondrial Cyt C release. The maladaptive UPR signalling in ER is facilitated via PERR, IRE1 receptors where the cells undergo apoptosis and inflammation. Aβ interacts with 20S proteosome and inhibits its functions such as degradation of the unfolded proteins. ATF6 is transported and processed in GC which acts as a transcription factor, transcribes the genes related to ERAD and XBP1. GC fragmentation is induced by the activation of several kinases which phosphorylates the GRASP65 and GM130. Autophagy and lysosome gene transcription is impeded via TFEB inhibition. On the other hand, the autophagy elongation is perturbed, as well as inhibition of autophagosome/endophagosome fusion with lysosome enhances its accumulation which drives cell death. In addition, Aβ ruptures the lysosome membrane, deacidify the niche and inhibits its hydrolases. All together the stress recovery mechanism is completely affected by the Aβ.

Several lines of evidence including cultured cells, animals and human brain slices reveal the relationship between the ER stress and Aβ. The most widely proposed connection is Ca^2+^ where the Aβ induced Ca^2+^ dysregulation triggers the ER stress mediated cell death. Analysis of AD brain revealed proteostasis dysfunction, Ca^2+^ dysregulation, and elevated level of molecular chaperones such as heat shock protein-27 (HSP27) and 78-kDa glucose-regulated protein or binding immunoglobulin protein (GRP78/BiP), the characteristic features of ER stress [161,162]. Interestingly, the FAD mutations in PS1 and PS2, and Aβ triggers inositol 1,4,5-trisphosphate receptor (IP3R) and ryanodine receptor (RyR) promoting release of Ca^2+^ ions from the ER, the early pathogenic sign in AD [163,164]. The detrimental role of Aβ is broadly studied in neuronal cells where the AβO increases the Ca^2+^ influx into the cells via stimulation of NMDA receptor (Figure 4) [165]. However, in cortical neurons, Aβ treatment prompted Ca^2+^ discharge from ER into the cytoplasm via IP3R and RyR, provoking ROS production, which in turn disrupts MMP enhancing the release of cytochrome C eventually leading to caspase-mediated apoptosis [166]. The crosstalk between the ER and mitochondria via MAMs play a significant role in the induction of apoptosis. The MAM, Mfn2 dimers restrains the tight junction between both the organelles. Sigma-1 receptor (Sig-1R) recognizes the ER-IP3R released Ca^2+^ concentrations facilitating its diffusion into the mitochondria [167]. During ER stress, the adaptive response from ER stimulates the increased mitochondrial metabolism and energy production. However, the maladaptive response from the ER impairs the mitochondria progression and triggers the cell death signalling which is directly proportional to the level of Ca^2+^ exchange between the two organelles [168,169]. The excessive ER Ca^2+^ discharge promotes the rapid accumulation of the toxic proteins including unfolded and misfolded proteins that leads to activation of UPR signalling. PERK activation and its signalling are engaged in the cognitive impairment of AD mice, where the increased p-eIF2α reduces the global protein synthesis inducing synaptic dysfunction and neurodegeneration. However, several reports suggest that Aβ treatment promotes ER stress. The APP/PS1 transgenic mice and Aβ treated SH-SY5Y cells showed enhanced level of GRP78, p-eIF2α, p-PERK, CHOP, and ATF-6 [170,171]. The E693Δ mutation in APP expresses high level of AβO, not fibrillation provoking ER stress and TGC dysfunction in cultured cells [172]. In the drosophila AD model, expression of XBP1 decreases the Aβ neurotoxicity where it inhibits the Aβ mediated overloading of the Ca^2+^ in the cytoplasm [173]. Multiple pieces of evidence reveal IRE1 mediated neurodegeneration in AD. IRE1 interaction with PS1 activates the proapoptotic pathway via JNK. A high level of JNK3 and phosphorylated JNK is observed in the post-mortem brain samples and cerebrospinal fluid (CSF) of AD patients, which is correlated with the Aβ42 levels [174]. A mechanism postulated that the IRE1 activates proapoptotic signaling via forming a complex between tumor necrosis factor receptor-associated factor 2 (TRAF2) and apoptosis signal-regulating kinase 1 (ASK1) that activates the various proteins and signalling pathways including NF-κB, JNK, caspase-12 and p38MAPK mediated CHOP [175,176,177]. Analysis of AD mice brain (Tg2576) samples reveal a substantial increase in the level XBP1 mRNA splicing in comparison with the age matched controls. The elevated level of XBP1 induces activation of CHOP, caspase cascade including caspase-3, 4 and 12 which pave the way to cell death [178]. The pathogenic role of IRE1-XBP1 is validated in various samples including the AD human tissue, IRE1 knockout (IRE1cKO) 5xFAD mice and XBP1 silenced Neuro2A cells. The human AD brains samples showed enhanced levels of p-IRE1 and spiced XBP1, whereas the healthy control brain samples reveal lower or undetectable levels of p-IRE1 and spiced XBP1. The IRE1cKO-5xFAD mice exhibits the characteristic of at least 50% reductions in the levels of the APP, Aβ monomers and Aβ plaques with improved learning and memory, and restoration of LTP in comparison with 5xFAD control. The level of APP is dramatically declined in the IRE1cKO-5xFAD mice, but not APP mRNA, which suggests that UPR controls the APP at post-translational level. Similarly, the XBP1 over expressed cells show increased APP expression, which is attenuated in the XBP1 silenced cells. Overall, XBP1 further elevates the expression of APP and production Aβ which facilitates the neurodegeneration by the maladaptive UPR (Figure 4) [179].

Bundles of literature evidence reveal relationship between the Aβ and ubiquitin-proteasome system (UPS). The E3 enzyme performs an important role in the ubiquitination process, which connects the Ub substrate protein and proteasome. The enzymes Parkin, HRD1 and UCHL-1 regulates the ERAD mechanisms including ubiquitination, ER membrane translocation, and proteasomal degradation [180]. The hippocampal and cortex regions of the AD patient showed co-residents of Aβ and parkin, which suggest that the parkin ubiquitinates the Aβ. However, the parkin over expressed AD mice showed Aβ-parkin ubiquitination, but the parkins expression was downregulated, and increased extra cellular plaque formation was observed, which indicates that the Ub-Aβ suppresses the parkins activity [181]. In the AD patient brain, the mRNA and protein expression studies revealed that the level of HRD1 is drastically decreased in comparison with healthy brain. Moreover, the biochemical analysis of Aβ is significantly increased in AD brain, which correlates with the HRD1 expression. On the other hand, the HRD1 over expressed SHSY5Y cells exhibits decreased expression of APP and Aβ production inside the ER. The immunocytochemistry analysis expresses that colocalization of HRD1 with APP in proline-rich sites promotes ubiquitination and degradation. The mutant and wild type HRD1 expressed HEK293 cells alters the APP ubiquitination, the wildtype HRD1 promotes the APP degradation whereas the mutant HRD1 elevates the APP expression and Aβ generation [35]. The UCHL-1 is a deubiquitinating enzyme that maintains the cellular ubiquitin process. In AD transgenic mice, downregulation of UCHL-1 was observed which, impaired the expression of BDNF/TrkB. Similarly, the Aβ treated cortical or hippocampal neurons showed downregulation of BDNF trafficking and signaling (including ERK5 activation and CREB-dependent gene regulation) due to the decreased level of UCHL-1, which was reversed by overexpression of UCHL-1 [182]. A report reveals that Aβ diffuses into the cytoplasm from the lumen of the ER where the Aβ is ruined by the proteasome and IDE [183]. In contrast, the proteasome inhibition by lactacystin decreases the Aβ degradation in the primary cortical and astrocyte cells. In addition, alteration in the ubiquitin-proteasome leads to the unusual accumulation of Aβ (Figure 4) [184]. The AD brain shows increased accumulation of Ub-protein complex, which results in proteasomal dysfunction induced by Aβ. It is hypothesised that the Aβ get into the active site of 20S proteasome subunits and inhibits its chymotrypsin-like activity. Further, Aβ aggregates acts as a competitive substrate of the chymotrypsin-like activity of 20S proteasome where the proteasomal functions get impaired elevating the level of AβO in the AD patients (Figure 4) [185]. On the other hand, the membralin is an ER protein with predicted transmembrane loops and does not have any domains. A recent genome wide association approach revealed that a 500bp single nucleotide polymorphism is linked with LOAD. Interactome network analysis discovers that the membralin is an ERAD component that retains homeostasis by degradation of membrane and pathological substrates including nicastrin [186]. However, the mRNA and protein expression of membralin is reduced in AD brain, where significant enhancement of γ-secretase activity was observed. The membralin shRNA treated mice brain and N2a cells showed downregulation of membralin in dentate gyrus region and elevated level of CHOP and XBP1. In addition, a significant rise in the amount of nicastrin and Aβ plaque load was noted [187].

## 6. Autophagy/Lysosomal Dysfunction

### 6.1. Autophagy Physiology

The cells undergo clearance of damaged organelles and unwanted protein aggregates via lysosome mediated degradation termed as autophagy. Autophagy supports the organelles to regulate its homeostasis and maintains the cellular nutrient level. Based on consignment transport the autophagy is broadly divided into three distinct groups such as micro-autophagy, chaperone-mediated autophagy (CMA), and macro-autophagy. Micro-autophagy involves direct invagination of the cytoplasmic portion by the lysosomes for degradation. CMA deals with the specific KFERQ sequence of the cytoplasmic proteins targeted by the Hsc 70 and its co-chaperones where this complex is transported to the lumen of the lysosome via membrane receptor lysosome associated membrane protein type 2A (LAMP-2A) for degradation. The p38-MAPK regulates the CMA process via phosphorylation and activation of LAMP-2A where inhibition of MAPK specifically inhibits the CMA [188,189]. Macro-autophagy (here referred as autophagy) is regulated in three steps, nucleation, elongation, and lysosomal degradation. In brief, autophagy nucleation is required for the Atg1/Unc-51-like kinase (ULK) complex to recruit the crucial proteins for autophagosome formation. Autophagy initiation is controlled by phosphorylation of ULK1 by the mammalian target of rapamycin complex 1 (mTORC1). Under nutrients rich condition the mTORC1 is recruited to lysosome membrane by Rag complex where the mTORC is activated by GTP-bound Rheb. The activated mTORC1 phosphorylates the ULK1 at Ser757, which suppresses the autophagy initiation as well as the transcription factor EB (TFEB). Under stress, mTORC1 becomes inactivated that results in activation of TFEB and AMPK mediated by galectins. The galectins interaction with the Rag complex inactivates the mTORC1 upon dissociation where the activated TFEB translocate into nucleus and activates the autophagy and lysosomal genes. In addition, AMPK activation phosphorylates the ULK1, which further phosphorylates the proteins FAK family kinase interacting protein of 200 kDa (FIP200) and the autophagy-related proteins ATG13 and ATG101. Further, ULK1 activates beclin1-vacuolar protein sorting 34 (VSP34) complex, which works as a Class III phosphatidylinositol 3-kinase (PI3KCIII) to generate phosphatidylinositol 3-phosphate (PI3P) that hires its binding proteins for the phagophore nucleation. The phagophore elongation depends on either ATG8 (LC3 and GABARAP) or ATG12 (ATG12 and ATG5) ubiquitin-like conjugation systems. The E1 (ATG7) and E2 (ATG10) ubiquitin ligases conjugates the ATG12 and ATG5, further ATG12-ATG5 binds to the ATG16L1 which primes the recruitment of microtubule associated proteins LC3. The ATG5-ATG12- ATG16L1 complex produces LC3-II that helps the extension and closure of the phagophore to become mature autophagosome. At last, the lysosome fusion with the autophagosome takes place where the lysosome hydrolases digest the contents of the autophagosome [190,191].

### 6.2. Autophagy Impairment by Aβ

Several lines of evidence prove that autophagy impairment plays a vital role in the neurodegeneration. In the AD brain, the abundant accumulation of autophagic vacuoles (AVs) in dystrophic dendrites illustrates impaired AVs maturation (Figure 4). Subsequently, the AVs act as a reservoir for Aβ, the purified AVs are enriched with β-CTF (C99) together with the components of γ- secretase. Further, the genesis of AD specific EVs containing MHC class-type markers where the disruption of autophagic proteins such as GABARAP and LAMP1 are the symptomatic appearance of AD patients which aids the AD clinical diagnostics and treatment [192]. In addition, the rapamycin-induced mTOR inhibition activates the autophagy where the γ- secretase complex translocates predominantly from the endosomes to AV [193,194]. Fedeli et al. uncovered that PS2 mutations impair autophagy through clogging the autophagosome-lysosome fusion process, which due to the lessened recruitment of GTPase RAB7 to autophagosome and altered Ca^2+^ homeostasis [195]. Beclin-1- an autophagy regulating protein is diminished in AD patients. Baclin-1 deleted mice exhibited reduction in autophagy and impaired lysosome, thereby accelerating intra-neuronal Aβ accumulation [196]. Beclin-1 stimulates degradation of the PM-APP and its metabolites via endosomes and endo-lysosome which is negatively regulated by AKT [197]. Autophagic proteins such as ATG5, ATG 12, and LC3 were discovered in association with the Aβ plaque in AD cells [198]. In the drosophila model, the ATG1, ATG8a, and ATG18 were downregulated depending on the age, subsequently the autophagy induction is decreased with increase in Aβ production [199]. Accretion of mutant APP and Aβ in the hippocampus cells of APP mice explicates the decreased level of autophagy protein (ATG5, LC3BL1, and LC3BII) [200]. The LC3-II is ambiguously increased along with accumulated Aβ, which indicates the induction of autophagy, whereas LC3-II lysosomal degradation is hindered. A prenylated protein Rab7 is vital for autophagy progression, which colocalizes with LC3-II, though the Aβ treatment has dropped its colocalization with LC3-II (Figure 4) [201]. Further, Aβ disturbs the functions of the dynein and kinesin which are essential for the axonal anterograde and retrograde transportation. Aβ within the AVs competitively impedes the pairing of dynein and snapin, and its complex, which is necessary for AVs cargo transportation towards the perinuclear space for lysosomal degradation. Aβ precisely interacts with the dynein, axonemal, intermediate chain (DNAIC) which clogs the organization of dynein-snapin motor-adaptor complexes [202,203]. Likewise, the dynein subunit dynactin-P50 expression is downregulated in AD brain with APOE mutations and the dynactin-P50 colocalized with Aβ plaques [204]. AβO inhibits the bidirectional axonal transport via the endogenous activation of casein kinase 2 (CK2) which is reversed by CK2 inhibitors. Both AβO and CK2 enhance the phosphorylation of kinesin-1 light chains (LLCs) which causes the kinesin-1 liberation from vesicular loads that perturb the fast-axonal transport [202]. Autophagy is transcriptionally regulated by the NRF2 (nuclear factor, erythroid 2 like 2) where it activates autophagic genes. NRF2 deficient mice along with APPV717 and MAPTP301L mutations exhibit elevated levels of APPV717 and MAPTP301L proteins and the expression P62, NDP52, ULK1, ATG5 and GABARAPL1 is reduced [205]. The reverse translational studies (man to mice) revealed that the NRF2 deficiency increases the p-tau and AβO, which renders significant rise in oxidative and inflammatory stress [206]. Furthermore, the increased BACE1 and mRNA stabilizing antisense (BACE-1-AS) is silenced by NRF2 by linking with antioxidant response elements, whereas the NRF2 deficiency upregulates the BACE-1 and BACE-1-AS expression and Aβ generation thereby enhancing the cognitive impairment [207]. Overall, the data suggest that the activation of NRF2 may act as a potential therapeutic target to reduce AD pathogenesis.

Lysosome plays a key role in maintaining the protein homeostasis of a cell where proteins are degraded in the heterogeneous compartments, the autolysososme or endolysosome. Several lines of evidence prove the lysosomal proteolytic failure could potentially cause the accumulation of intermediate autophagy compartments autophagososmes, autolysosome and AVs in the neurons [193]. Mutations in PS1 or deletion of PS1 aggravate the autophagy pathology. Apart from participating in γ-secretase complex, the PS1 is involved in lysosome acidification and accelerates the autophagosome-lysosome fusion [208]. The reports explore that PS1 acts as a chaperon for v-ATPase (vacuolar ATPase) where it mediates N-glycosylation of V0a1 subunit which facilitates the lysosomal acidification through pumping the protons whereas the PS1^-/-^ impairs the lysosomal acidification [209]. Coen et al., postulated an alternative hypothesis that the PS1^-/-^ cells exhibit the lysosomal Ca^2+^ efflux which may impair the lysosomal fusion capacity [210]. Immunogold electron microscopy studies reveal the coresidents of mature nicastrin, PS-1, and APP with lysosomal associated membrane protein-1 (cAMP-1) in lysosomal membrane where the Aβ production occurs within the lysosome [57]. The intracellular Aβ is unaffected by proteases which last at least 48 h in the cultured neuron. Aβ exposure in neuronal cells evokes the oxidative stress which disrupts the membrane proton gradient via damaging the lysosomal membrane which is blocked by treatment with either methylamine or n-propyl gallate that prevents the lysosomal leakage [211]. Several reports showed that the function of lysosomal enzymes was diminished in the AD patients impairing the toxic Aβ clearance and autophagic functions (Figure 4) [212]. The acidic environment accelerates the lysosomal enzyme activity, failure of which leads to protein clearance deficiency. A master protein TFEB regulates the functions of autophagy and lysosomes via manifesting the lysosomal enzymes and producing the membrane proteins upon translocating to the nucleus. Further, osteopetrosis-associated transmembrane protein 1 (OSTM1) in collaboration with chloride channel 7 (CLCN7) controls the lysosomal pH and Aβ clearance, which is closely regulated by the nucleus translocation of the TFEB. Aβ treated microglial cells showed dose dependent nuclear diminution of TFEB, with parallel increase in cytoplasm. Similarly, OSTM1 expression was significantly downregulated along with poor lysosome acidification, which suggest that inhibition of the TFEB nuclear translocation mediates the lysosomal dysfunction [213]. A lysosomal aspartic protease, Cathepsin D is responsible for the degradation of aged and toxic proteins including Aβ. Cathepsin D zymogen is activated in an acidic compartment by cleaving the pro-peptide. Cathepsin D is highly expressed in the AD brain as an initial event. APP-C99 impairs the lysosomal functions by increasing the lysosomal pH and inactivating the cathepsin D and other hydrolases, while silencing APP or inhibiting BACE-1 rescues the cathepsin functions [214]. Aβ treated neuroblastoma cells exhibit cellular alterations as observed in AD such as external administration of Aβ was initially detected in the clathrin-positive organelles, and later in lysosomes. Further, cellular Aβ uptake facilitates the formation of autophagosome and destruction of lysosomal membranes, leaking its contents into the cytoplasm. Notably, the cells showed enhanced autophagosomes invagination in the nuclear envelopes, which showed the connection between autophagosomes accumulation and cell death [215]. Gowrishanker et al. demonstrated that Aβ accumulated vesicles is dwelled within the swollen axons of the neurons, where the LAMP1 staining explores the recruitment of LAMP1 by autophagosome fused late endosome for maturation to lysosomes. These lysosomes have Aβ plaques within its compartment where it contains very lower concentrations of cathepsins B, D, and L (as well as AEP) than the lysosomes present in the soma of a cell. Further, the enhanced BACE1 expression within the swollen axons indicates that the defective axonal lysosome transport and maturation might boost up the Aβ production [216].

## 7. Aβ-Accelerated Golgi Fragmentation

### 7.1. APP Processing in GC

GC is a warehouse of cells where the lipids and proteins are processed and sorted as well as transported to various destinations. In addition, GC is playing a vital role in ion homeostasis, apoptosis, and stress sensing in mammals. GC is assembled with closely connected parallel cisterna known as Golgi stacks which are laterally associated by tubules forming a continuous ribbon that helps for the protein processing and transporting to its targets. The Golgi stacks has two distinct sides, cis- and trans-, where the cis-Golgi network located near ER for the entry and processing of substances and TGN is inhabited near the PM to deliver the products to its destination. This transportation amongst these organelles is mediated by the coat vesicles. GC plays an important role in processing the synthesized APP and BACE-1 and transportation through the secretory pathway. Tan et al. discovered the transportation route of BACE-1 which is different from APP transportation. In primary neurons and HeLa cells, BACE-1 transportation is facilitated by the AP-1 and Arf1/4 dependent manner, as well as BACE-1, which is recycled through the endosomal pathway. Inhibition of BACE-1 transportation increases the amyloidogenic cleavage of APP and Aβ production [217]. On other hand, the APP transportation is facilitated via recruitment of Arl5b-AP4 but not AP-1 to the GC. The perturbation in either Arl5b/AP4 raises the APP accumulation but not BACE-1 which indicates the diverted transportation of APP and BACE-1 [218]. Despite the literature proving that APP processing occurs in the endosomes, Choy et al. investigated the post-endocytic trafficking events in Aβ through the RNAi technique. HRS and TSG101 reduction present the APP at early endosomes and reduces the Aβ production. In opposition, diminution of CHMP6 and VPS4 rerouted the APP from endosome to GC for APP processing; where VSP35 mediated retrograde transport is needed for Aβ production. It has been suggested that the GC may be one of the intracellular sites for Aβ production [59].

### 7.2. Golgi Fragmentation by Aβ

The Golgi stack and ribbon organization are maintained by a complex molecular system such as Golgi matrix proteins (GRASP55, GASP65, GM130, Golgin-45, Golgin-84, Golgin-160), tethering proteins (p115/SNARE protein), microtubule related motor proteins, signaling proteins and proteins related to pH and Ca^2+^ homeostasis [219]. Under physiological conditions, the Golgi fragmentation is crucial to begin the mitosis, which is a highly organized process. Several kinases such as Cdc2, GSK3β, RAF/MEK1/ERK1c, Plk1, and Plk3 phosphorylates number of Golgi proteins, which facilitate the Golgi dispersion for mitosis. After cytokinesis, the fragmented Golgi reunites and serves its normal functions. However, under the pathological conditions such as apoptosis, the Golgi fragmentation is irreversible due to the caspase-mediated proteolytic cleavage of several Golgi proteins including golgin, t-SNARE syntaxin 5 and GRASP-65 [220]. Notably, many neurodegenerative diseases such as Alzheimer’s and Parkinson’s exhibit the Golgi fragmentation as a common hallmark. However, the Golgi fragmentation in neurodegeneration is an earlier irreversible process, which triggers the apoptosis. Several reports from both AD patients and animal models depict the Golgi fragmentation as a consequence of ER stress and oxidative/nitrosative insults or excitotoxins. In the AD brain, Aβ deposition alters the morphology of GC by dropping the mitochondrial membrane potential and release of cytochrome c in the cytoplasm. The immunogold-electron microscopic studies on AD mice (APP-PS1) model and Aβ-treated BV-2 cells showed Golgi cisternae fragmentation mediated by the COPI depletion which affects the intra-Golgi transport through Aβ deposition [221]. The Golgi morphological defects were observed in both AD animal and cells models such as Golgi fragmentation. The GC of AD mice (APP_Swe_/PS1_∆E9_) hippocampal and cortical tissues were fragmented with swollen cisternae, but in wild type mice normal ribbon-like organization was observed [222]. Similarly, APP_Swe_/PS1_∆E9_ overexpressed CHO cells reveal Golgi fragmentation, which decreases the APP trafficking and increases the Aβ production [222].

The molecular view of Aβ mediated Golgi fragmentation is reported by Joshi et al. where the AD mice and APP_swe_/PS1_ΔE9_ transfected cells exhibit an elevated amount of Aβ. Aβ induced CDK-5 activation facilitates the Golgi fragmentation via phosphorylation of Golgi proteins such as GRASP65 which is rescued by CDK-5 inhibition as well as expression of non-phosphorylatable GRASP65 mutants that also reduces Aβ production [223]. However, Golgi fragmentation in the Aβ treated cells is reversible upon removal of Aβ treated cell culture media. The electron microscope analysis portraits that the GC ribbons are disconnected with shorter and low numbers of cisternae per stack, with numerous vesicles adjacent to each stack. The relationship between the CDK5 and Golgi fragmentation in AD is explored upon the Aβ and glutamate treatment in the PC12, SHSY5Y cells where the cells undergo Golgi fragmentation via phosphorylation of GM130 which leads to cell death. GM130 acts as a substrate for CDK5 which impedes the binding of GM130 and vesicle docking protein p115 [224]. Furthermore, the CDK5 induces the p-tau and formation intracellular NFT. Reports indicates that siRNA mediated Golgin-84 diminution in HEK293 cells induce Golgi fragmentation and p-tau is mediated by the CDK5 and ERK kinases which indicates that depletion of Golgin-84 activates the CDK5 and ERK kinases [225]. On the other hand, GSK3β is activated by the Aβ which indicates that the GSK3β activation could develop a feedforward loop that promotes further APP amyloidogenic processing via activation of BACE-1 [222]. Further, GSK3β facilitates p-tau, which disturbs the microtubule network inducing Golgi fragmentation and neuronal malfunction [226]. In addition, many reports indicate that the JNK activity is highly linked with AD progression through higher Aβ generation and NFT formation. JNK2 plays a vital role in the separation of Golgi stacks via phosphorylation of GRASP65, whereas RNAi, or JNK inhibitors mediated JNK2 inhibition can restore the Golgi ribbon [227]. On other hand, the Golgi fragmentation in progressive motor neuronopathy mice lack TBCE and TBCE-depleted motor neurons with defective Golgi engaged microtubules and decreased COPI vesicles diminishing the recruitment of p115/GM130 proteins and SNARE mediated vesicle fusion. siRNA mediated SNARE Syx5 inhibition facilitates the Golgi fragmentation, similar to the reports of the Golgi fragmentation in the AD brain cells (Figure 4). In contrast, overexpression of Syx5 shows accretion of APP in the ER, which restrains the APP processing towards Aβ [228,229]. Recently, Suga et al. explored that Syx5 works as a stress rescuing component that involves in the neuronal cell survival. In detail, the apoptosis inducers decrease the expression of Syx5, whereas the ER stress inducers increased the levels of Syx5 and Bet1 protein expression. Syx5 deletion during apoptosis or ER stress causes the cell highly vulnerable. Further, Golgi stress increased the expression of Syx5 and concurrently reduced the Aβ production. These data suggest that the Syx5 is a common stress reducing agent for both ER and Golgi, where the Syx5 reduction in AD is due to the Aβ mediated inhibition of stress response [230]. Hence, protecting the Golgi structure and function denotes a new comprehension to reduce the Aβ generation and its toxicity.

## 8. Aβ in Gene Regulation

### 8.1. Aβ as Transcription Factor

Nucleus is a global control center of a cell where the genetic information is replicated and transcribed, which regulates the cellular behaviours. Bundles of reports confirm that as observed in other organelles, Aβ translocate into the nucleus, interacts with several nuclear proteins, and alters the gene expression. The soluble Aβ translocation into the nucleus is confirmed multiple techniques such as chemical testing of nuclear fragments, biotin labelled Aβ confocal imaging and transmission electron microscopic analysis of cultured cells. Possibly, the Aβ is passed directly into the nucleus through the channel-like pores. Remarkably, this study also explores the involvement of Aβ in nuclear signaling, the ChIP assay shows the specific interaction of Aβ with the LRP1 and KAI1 promotors, which potentially decreases the mRNA expression of the candidate genes [231]. Both LRP1 and KAI1 protect the neuronal cells against Aβ neurotoxicity. Aβ acts as a putative transcription factor for AD linked genes such as APOE, APP and BACE1. The electrophoretic mobility assay reveals that Aβ is precisely docked with the Aβ interacting domain (AβID) of the nucleus with the consensus of “KGGRKTGGGG” where any mutation in it, the peptide-DNA interaction is neglected [232]. Hence, Aβ itself act as a transcription factor and can control the transcription of candidate genes. The Aβ-chromatin interaction is discovered in the polymorphic APP-promotor CAT fusion clones transfected PC12 cells using ChIP assay. This transfected cells when supplemented with Aβ elucidates the DNA sequence specific response where it regulates its own amyloidogenic proteins such as APP and BACE-1 that induces more Aβ production [233]. Similarly, the transcriptional regulation of Aβ is validated upon studying several genes including (I) the amyloidogenic genes such as ADAM10, BACE1, PS1, PS2, Nicastrin and APP, (II) AD risk genes APOE and TREM2, (III) learning and memory factors genes such as NMDAR and PKC zeta, (IV) kinases which contribute for p-tau including GSK3α, GSK3β and Cdk5 and (V) enzyme 1α-hydroxylase (1αOHase). The qRT-PCR analysis explores the upregulation of amyloidogenic and p-tau related genes, which generate toxic Aβ and p-tau, and the downregulation of NMDARs, ApoE, Trem2, and 1αOHase genes [234]. DNA microarray analysis of Aβ treated neuroblastoma cells explore the upregulation of the insulin-like growth factor binding proteins 3 and 5 (IGFBP3/5). The qRT-PCR results confirm the above finding that the expression level of IGFBP3/5 is two-fold increased. Literatures indicate that IGFBP3/5 contributes to p-Tau. Further, the immunohistochemistry studies support these data illustrating higher expression of IGFBPswere observed in the hippocampal and cortical neurons. Further, the proteomic studies CSF of human AD, illustrates the appearance of elevated amount of IGFBP. These data suggest that both transcriptional and translational regulation of IGFBP by Aβ could be an early biomarker for AD [235]. In addition to Aβ, several reports implicate that the secretase cleaved fragments undergo nuclear translocation and controls the transcription regulation. In an AD patient’s brain, the γ-secretase cleaved ~6 kDa CTF- APP-like protein 2 translocate to nucleus and interacts with CP2 transcription factor where it upregulates the expression of GSK3β which contribute variety of pathological events for neurodegeneration [236]. Similarly, the γ-secretase cleaved APP-CTF in cytoplasm binds with an adapter protein Fe65, the confocal and FRET analysis discloses colocalization of GFP-APP-CTP and myc-Fe65 and translocation to the nucleus. Taken together, the APP-CT-Fe65 complex can potentially modify the transcription of the cells [65].

### 8.2. Telomerase, Spliceosome Inhibition and DNA Methylation by Aβ

Several reports discovered the relationship between the telomerase and the AD pathology where the shortened telomerase involved in AD progression whereas the increased telomerase activity protects the neurons from the neurotoxic aggregates [237,238,239,240]. The cellular senescence also known as DNA damage response (DDR) is a prime factor of age-linked diseases. In the sight of DNA damage, the DDR coordinate the DNA damage through cell cycle arrest until the DNA damage is recovered, also DDR facilitates the perpetual growth arrest if the cells are failed to repair the damage [241]. Increasing evidence shows that the telomeres are engaged in development of neurodegeneration including AD [238,239]. In tissues and peripheral blood cells preferentially aged patient’s leucocyte, the telomere length is highly associated with AD risk and cognitive deficits. Few studies showed contrariety data in the telomerase length; however, numerous reports confirm that the leucocyte in reference to the age showed the excessive telomerase loss leading to AD development [242]. Recently, Wang et al. discovered that the AβO potentially inhibited the telomerase activity through interacting with DNA-RNA templates and RNA templates of telomerase and blocking the telomeric DNA elongation [240]. In addition, the Aβ colocalized telomere is also observed as a cause of telomerase inhibition. In contrast, overexpression of catalytic subunit of the telomerase decreases the Aβ induced cell apoptosis, which might be capable of defending the age-related neurodegeneration [237]. On the other hand, the epigenetic regulation including DNA methylation and histone modification controls the gene expression. DNA methylation obstructs the transcription factors binding with the DNA via transferring the methyl group to the cytosine CpG dinucleotides, which is catalysed by specific DNA methyltransferases. DNA methylation plays an important role in gene silencing/inactivation. The AD brain shows loss of DNA methylation through the increased concentrations of S-adenosylhomocysteine, a potential inhibitor of methyltransferase. Induction of DNA hypomethylation in the promotors of the AD associated genes including APP, PS1 and BACE1 accelerates abnormal expression of these genes, which leads to increased production and accumulation of Aβ [243]. Aβ-treated samples are digested with a methylation-sensitive (HpaII) or a methylation-insensitive (MspI) restriction endonuclease for the DNA microarray analysis. The results showed significant methylation changes in the genomic loci with highly enriched cell-fate genes, which control the apoptosis and neuronal differentiation involved in potentially inducing the brain contraction and memory deficits in AD [244]. The AD brain cortex and AD patient’s lymphocytes are analysed for epigenetic alterations at the promotor regions of several genes including PS1, and APOE. The PS1 is usually hypomethylated which triggers unusual Aβ generation, whereas the APOE has a bimodal structure where at most it is found in a hypermethylated state. Hence, the data suggest that the concurrent manifestation of both hyper- and hypo-methylation could potentially contribute for AD progression [245]. The Aβ mediated epigenetic regulation including DNA methylation/demethylation of a specific promotor is widely studied. The Aβ induced oxidative stress is a prime causative of DNA hypermethylation in an aging brain. Chen et al. discovered that the Aβ suppresses the neprilysin (NEP) promotors via DNA methylation [246]. The HPLC and methylation specific PCR studies of the Aβ treated endothelial cells showed increased NEP methylation which suppresses the NEP mRNA and protein expression. The NEP is a zinc metalloproteinase, which facilitates Aβ clearance in AD mice. Thus, the results suggest that the DNA methylation of NEP is a consequence of Aβ accumulation. In addition, the modification in the alternative splicing is highly linked with the AD progression. The LOAD is characterized with U1 small nuclear ribonucleoprotein (snRNP) tangle-like deposition due to the aberrant genetic mutations in PS1and APP, which results in unusual APP processing leading to formation of snRNP aggregates [247]. Aβ treated neuroblastoma cells were subjected to the proteomic analysis to expound the early events of AD pathogenesis. Remarkably, the bioinformatics results imply the downregulation of ribosomal biogenesis and splicing process. Further, Western blot analysis explicated the downregulation of each splicing steps facilitating the downregulation of every subunit of the spliceosome. These results suggest that the spliceosome dysfunction is a consequence of Aβ deposition. Overall, Aβ is acting as gene regulating factor upon interacting with telomerase/telomeres, epigenetic and transcriptional regulation, and aberrant spliceosome function which contributes to high risk of AD progression.

## 9. Signalling Mechanism of Aβ Leading to Memory Impairment and Cell Death

### 9.1. Receptor Mediated Long Term Potentiation Inhibition

It is well known that the extracellular Aβ interacts with the surface of the brain cells where the Aβ activates several signalling mechanisms unusually that triggers the cells either to survive or die. As a good sign, Aβ interaction with the receptors such as low-density lipoprotein receptor-related protein 1 (LRP1), low-density lipoprotein receptor (LDLR), scavenger receptors A1 and A2 (SCARA1 and SCARA2) facilitates the Aβ uptake and clearance [248,249,250]. In addition, Aβ binding to the macrophage receptor with collagenous structure (MARCO) activates the extracellular signal regulated kinase 1/2 (ERK1/2) signalling pathway, which reduces inflammation [251]. In contrast, various receptors on synapse show toxic effect, causing the synaptic dysfunction and neurodegeneration. The receptors of Aβ transduce the specific intracellular changes via activating the extracellular factors either directly or association with other molecules. The AMPARs and NMDAR are the ligand gated ionotropic glutamate receptors, and the mGluRs regulates the learning and memory via LTP and long-term depression (LTD) at excitatory synapses. The lower synaptic signal activates AMPARs, and the stronger synaptic signals unblock the NMDARs, which results in increased number of AMPARs on the post synaptic membrane [252]. The increased LTP was observed when there is over-expression of AMPAR at postsynaptic membrane; in contrast, some reports reveal that elimination of AMPARs increases the LTD [253,254]. The role of AMPARs in AD is still unclear, however, the results suggest that the AMPARs are downregulated during the preliminary stage of AD. Aβ25-35 treated rat embryonic hippocampal cells showed elevated level of caspase activity, which leads to enzymatic degradation of the AMPAR not NMDAR [255]. Apart from the enzymatic cleavage, Aβ directly interacts with AMPAR and modulates it functions. Iontophoretically exposed aggregated Aβ1–42 on the hippocampal CA1 neurons reduces the AMPA-induced neuronal firing, but NMDA-evoked neuronal firing was enhanced, which suggest that the LTP disruption and attenuation of field excitatory postsynaptic potential (fEPSP) [256]. On the other hand, lower synaptic stimuli trigger either NMDARs to generate NMDA-mediated LTD or mGluRs to make mGluR-dependent LTD, which prompt the removal of postsynaptic AMPAR [257]. Patient-specific human iPSCs derived neurons produced Aβ exhibits synaptotoxic mediated cell death showing impaired axonal vesicle clusters, postsynaptic loss of AMPAR and rise in Aβ mediated tau phosphorylation [258]. Numerous protein kinases and phosphatases play a vital role in generation of LTP and LTD. AβO interrupts the postsynaptic Ca^2+^signalling through increasing the accessibility of glutamate molecules to the NMDAR. The enhanced activation of NMDAR causes abnormal redox reactions as well as increased Ca^2+^ influx into neurons that activate the Ca^2+^-dependent protein phosphatase calcineurin/PP2B and protein phosphatase 2A (PP2A) [259]. Activation of calcineurin further activates or deactivate the target proteins via dephosphorylation. The synaptic dysfunction is mediated by the surface removal and endocytosis of AMPAR. Aβ-stimulated AMPAR endocytosis is reliant on the activation of calcineurin/PP2B, which is mediated by downregulation of CaMKII [260,261]. Like AMPARs, Aβ also can prompt NMDARs internalization, which is mediated by dephosphorylation of GluN2B (NMDAR subunit) of p-Tyr1472 in the striatal-enriched protein tyrosine phosphatase (STEP) [262]. AβO downregulates the glutamate transporters EAAT1 and EAAT2 of glial cells, which disrupts the glutamate uptake causing the glutamate overflow at synaptic cleft that over-activates the GluN2B [263].

NMDAR and AMPAR interactions with post-synaptic density scaffolding protein (PSD-95) at post-synaptic membrane regulate the protein assembly and neural plasticity [264]. Aβ exposed cortical neurons exhibits CDK5 and NMDAR mediated reduction on PSD-95 that leads to synaptic dysfunction and surface AMPAR removal [265]. Co-immunoprecipitation studies on human post-mortem AD brain and AβO treated murine neurons shows that the Aβ directly interacts with PSD-95 at post synaptic membrane causing synaptic loss [266]. Cellular PrPC has high affinity with AβO, such molecular associations of AβO-PrPC found only in the AD brains, but not control brains [267]. AβO-PrPC inhibits the hippocampal LTP that manifests the memory deficit in an AD mouse model [268]. AβO bound PrPC influences the activation of Fyn kinase that in switches the GluN2B phosphorylation, resulting in NMDARs surface removal. The AβO-PrPC complex demands both mGluR5 and LRP1 co-receptors to activate the Fyn [269,270]. In addition, the Fyn activation steers on tau phosphorylation [271]. PrPC resides at cholesterol- and sphingolipid-abundant, detergent-resistant lipid rafts. The saturated acyl chains of glycosylphosphatidylinositol trigger the N-terminal signal interaction with the heparan sulfate proteoglycan, glypican-1 [272]. PrPC knockout or anti-PrPC antibodies rescues the AβO-stimulated synaptic dysfunction and spatial memory, which indicates that the PrPC play a crucial role on AD pathogenesis [267]. In addition, the AβO activates α7-nAChR which increases the presynaptic Ca^2+^ level and disrupts the rafts by cholesterol depletion [273]. AβO activated α7-nAChR causes the elevated cytosolic Ca^2+^, calcineurin activation and dephosphorylation and activation of STEP61. The enhanced STEP61 inactivates Fyn and lowers the NMDAR exocytosis because of the GluN2B dephosphorylation mediated NMDAR internalization [261]. Likewise, AβO binds to the receptor tyrosine kinase EphB2 resulting in its degradation that causes reduction in NMDA receptor subunits such as GluN2B, which leads to impairment in NMDAR-mediated synaptic activity and cognitive function. In opposite, the EphB2 overexpressed AD Tg mice reverses the deficits of NMDAR-dependent LTP and cognitive impairments [274]. Further, Ephrin A4 (EphA4) was discovered as a putative Aβ receptor. Aβ mediated EphA4 activation leads to repression of LTP and spine loss in AD transgenic mice where the EphA4 shRNA or EphA4 inhibitors/antagonists inhibits these deficits [275,276]. Overall, the Aβ interaction with various receptors stimulates the neurotoxicity via NMDAR (Figure 2).

### 9.2. Receptor Mediated Cells Death Induced by Aβ

Ligand binding cell surface death receptors (DR) are the tumour necrosis factor (TNF) gene superfamily receptors that confer caspase mediated death pathway. DR contains cysteine rich extracellular domain and intra cellular death domain. The receptors including TNF receptor 1 (TNFR1), Fas receptor (FasR), TRAIL receptor 1 and 2 (TRAIL-R1 and R2), p75NTR and lymphoid cell specific receptors CD30, CD40 and CD27 facilitating the death signaling [277]. Interestingly, the Aβ has high affinity with these receptors and activates the apoptotic pathway. Ivins et al. hypothesised that Aβ may activate the Fas/TNFR mediated apoptosis signalling. The Aβ treated hippocampal neurons exhibit recruitment of caspase-8 and FADD proteins during the apoptotic event, which was prevented by the pre-treatment of caspase-8 specific inhibitor IETD-fmk and viral mediated dominant negative FADD gene delivery. Ivins and colleagues concluded that both the caspase-8 and FADD requirement in apoptosis support that the cell death might be initiated through Fas/TNFR family receptors upon interaction with Aβ (Figure 2) [278]. The vascular Aβ mediated extrinsic apoptotic signalling mechanism discovered using the human brain microvascular endothelial cells treated Aβ40 or its vasculotropic variants E22Q or L34V. The apoptotic cell death facilitated via binding of AβO with the (TRAIL) death receptors DR4 and DR5 followed by the activation of caspase-8 and caspase-9. Further, the caspase-8 inhibitor FLICE-like inhibitory protein (cFLIP) downregulated the mitochondrial path associated with the BH3-interacting domain death agonist (BID) cleavage. DR4 and DR5 up-regulation and co-localization with AβO indicate the receptor specific interaction, which was attenuated upon RNA silencing of both DR4 and DR5 [279].

Numerous reports discovered that the p75NTR, a nerve growth factor (NGF) receptor mediated cell death. The p75NTR is structurally similar to the p55 TNF and Fas receptors. Alteration in the p75NTR expression promotes cell death or survival, a decreased p75NTR increases cell survival whereas increased p75NTR induces apoptosis by silencing Trk-mediated survival signals. On the other hand, Aβ mediated cell death via p75NTR is altered by the NGF, the NGF binding instead of Aβ inhibits the p75NTR death signalling, however, the Aβ-p75NTR induced death is found in PC12 cells [280], NIH 3T3 cells [281], human neuroblastoma cells [282] and hippocampal neurons [283]. Aβ exposed mutant (p75NTR^−/−^) mice reveals the least cell death on the hippocampus compared with wild-type mice [284]. Aβ facilitates the apoptosis in cultured neurons through the activation of JNK–c-Jun–Fas ligand–Fas pathway [284]. Knowles et al. investigated the AβO interaction on the surface of p75NTR using fluorescence resonance energy transfer (FRET)-imaging technique. The role of p75NTR in Aβ-induced neuronal death and c-Jun expression is validated using p75NTR^−/−^ mutant mice derived neuronal cultures and p75NTR^−/−^ AD mice model. The results reveal that neurodegeneration through p75NTR requires AβO interaction on the surface domain of p75NTR [285]. A high level of Ca^2+^ inflow is reported as an important factor for AD, which is mediated via upregulated L-type Ca^2+^ channel in AD mice not in wild type mice. The overexpression of p75NTR prevented the Ca^2+^ channel current, but Aβ1-42 treatment significantly increased the Ca^2+^ channel current, due to the blockage or decreasing expression of p75NTR. The Aβ1-42 induced Ca^2+^ channel current activation is removed when the p75NTR expression is dropped (Figure 2) [286]. The high mobility group box 1 (HMGB1) acts as a proinflammatory mediator and it activates the inflammatory response via docking RAGE and Toll-like receptor 4 (TLR-4). The RAGE entails significantly in neurodegeneration by the action of several signalling moieties such as CaMK-β-AMPK, the RAGE/(ERK1/2), GSK-3β, and NF-κB, which directs the Aβ and p-tau pathology [287,288,289] while the TLR-4 acts as an immune receptor elucidating the immune response.

## 10. Inflammation a Central Mechanism in AD

**Figure 5:**
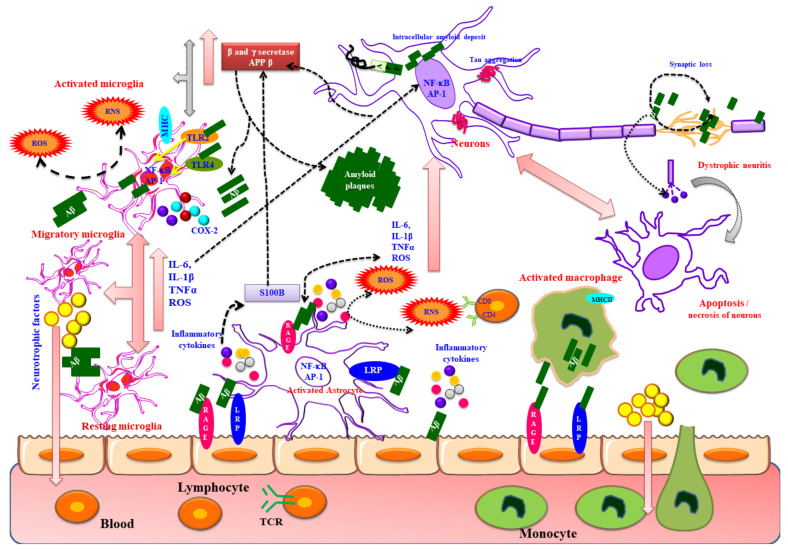
Neuroinflammation in the pathogenesis of Alzheimer’s disease: Aβ activates microglial cells via TLR and RAGE receptors which stimulates the NF-κB and AP-1 transcriptional factors leading to release of inflammatory cytokines (IL-1, IL-6, TNFα), ROS and RNS inducing oxidative/nitrosative stress mediated neuronal damage. Inflammatory cytokines stimulate astrocytes leading to amplification of inflammatory signals inducing neurotoxic effect. Chemokines also attracts peripheral immune cells towards amyloid plaque exacerbating inflammatory response.

Extensive research on pathogenesis leading to AD revealed that gap exists in between core pathologies, Aβ plaques and NFT in understanding AD pathogenesis. Paramount evidence indicated the existence of inflammatory markers in the brain of AD patients and preclinical AD model system, other than the neuropathological hallmarks senile plaques and PHF, indicating that inflammation acts as interlink between early lesion senile plaques and the later lesion NFT in AD pathogenesis [290]. Inflammation acts as double-edged sword, in a healthy brain, as acute inflammation plays the role of defense mechanism against various infection, toxin and injury clearing the invading pathogen or injurious agent. On the other hand, imbalance between pro and anti-inflammatory mediators due to Aβ accumulation in AD leads to chronic inflammation, characterized by the activation of microglial cells, which was observed to be accumulated around Aβ plaques in AD brain and transgenic animal models [291]. Microglial cells and astrocytes in its activated state release mediators of inflammation-like cytokines, chemokines, complements, monocyte chemoattractant, ROS and prostaglandins, etc., disrupting the balance between the normal neurophysiologic conditions associated with cognition, learning and memory. Inflammatory mediators activate more glial cells and astrocytes to release cytokines, which promotes the migration of monocytes and lymphocytes across the BBB towards Aβ accumulated site in brain of AD individuals triggering inflammatory response (Figure 5) [292,293]. Initially, constant inflammatory response was considered as causative for neuronal loss in AD patients, later substantial evidence revealed that persistent immune response facilitates and exacerbate both Aβ and NFT pathologies. Hence, inflammation is considered as a driving force, which induces or accelerates the pathogenesis of AD. Epidemiological studies revealed the linkage between the polymorphisms in the immune molecule involved in AD and the role of nonsteroidal anti-inflammatory drugs in attenuating the incidence of AD. The degree of inflammatory response depends on the level of Aβ, tau and ubiquitin and APOE ε4 as observed in AD sub types. McGeers et al. observed enhanced expression of HLA-DR (human leukocyte antigen, antigen D related) a Class II major histocompatibility complex (MHC) in microglial cells around the senile plaques [294]. Further, Giometto et al. revealed the presence of high level of complement and acute phase proteins in AD blood sample depicting the fact that both immune and inflammatory response synergistically triggers AD pathogenesis [295]. Scientific evidence on the use of anti-inflammatory drugs for the treatment of rheumatoid arthritis, in transgenic mice and human showed convincing results on reduction in AD pathogenesis revealing the fact that inflammation play key role in AD pathogenesis [296]. Microglia on chronic activation produces several proinflammatory mediators such as ROS, RNS and chemokines. Increase in the level of interleukin 1 (IL-1) enhancing the level of cerebral Aβ deposit was observed in deceased patient affected by head trauma, illustrating the facts that IL-1 promotes amyloidogenic processing of APP enhancing the level of Aβ peptide.

IL-1β enhances the release of IL-6 which activates CdK5 inducing hyperphosphorylation of tau [297]. These reports reveal that incidence of neuroinflammation occurs before the neuropathological hall marks aggravating Aβ load and hyperphosphorylation of tau protein, which in turn further activates the inflammatory pathway revealing the inter-relationship between these apparently contrasting core pathologies leading to AD (Figure 5).

###  10.1. Cellular Mediators Involved in Neuroinflammation

#### 10.1.1. Microglial Cells

Microglial cells are the specialized macrophages found in CNS which plays a vital role in restoring the brain homeostasis via inflammatory response, phagocytosis of Aβ plaques and NFT. Microglial cells in the resting state exist in ramified morphology portrayed with small cell body, which interacts with neurons and other glial cells via signaling mechanism through numerous receptors for neurotransmitters, cytokines maintaining the neurons healthy [298]. Microglia interacts with Aβ peptide through several receptors such as scavenger receptors (SR-SCARA-1, MARCO, SCARB-1, CD36 and RAGE); G protein-coupled receptors (GPCRs- formyl peptide receptor 2 (FPR2) and chemokine-like receptor 1 (CMKLR1)), and toll-like receptors (TLRs- TLR2, TLR4, and the co-receptor CD14). Receptors SCARA-1, SCARB-1, MARCO and CMKLR1 interact with Aβ promoting its cellular uptake, during which RAGE activates microglial cells to release proinflammatory molecules, while other receptors such as TLR, CD36 and FPR2 exhibit dual functions (Table 1) [299]. In early AD pathogenesis, Aβ peptide acts as primary driver triggering microglial cells towards plaques and provokes phagocytosis of Aβ peptide, but on prolonged activation results in exacerbation of AD pathology. Overall, neuroinflammation in AD is caused by microglial priming on interaction of Aβ peptide with receptors (SR1, GPCR, TLRs). Aβ fibrils recognizes the complex CD36-α6β1-CD47 leading to generation of ROS, providing signal for heterodimerization of TLR4-TLR6 transmitting signal for activation of NLRP3 a component of inflammasomes in microglial cells. Inflammasomes is an intracellular multiprotein complex composed of NLR family pyrin domain containing three (NLRP3), apoptosis-associated speck-like protein containing a caspase-recruitment domain (ASC) and procaspase- 1, which acts as first line of defense. Inflammasomes acts as platform for activation of caspase-1, which activates cytokines IL-1β and IL-18 the key mediators of inflammation [300]. CD36 promotes entry of Aβ into the lysosome inducing destabilization, dysfunction of lysosomes with consequent release of cathepsin B into cytosol. Cathepsin B induces NLRP3 dependent caspase-1 activation of IL-1β and IL-18, which in turn triggers the release of several chemokines (IL-1, IL-18 and TNF-α), chemotactic mediators stimulating nuclear factor-kappa-B (NFκB) dependent pathway [301,302]. Cytokines and chemokines exacerbate Aβ accumulation leading to activation of microglial cells enhancing the production of proinflammatory mediators provoking neurodegeneration in cyclic manner (Figure 5) [303]. Aβ peptide promoted activation of microglial cells, releasing proinflammatory mediators which in turn induced microgliosis and astrogliosis decreasing the efficiency of microglial cells to phagocytize Aβ peptide, reduction in Aβ degrading enzyme affecting the clearance of Aβ peptide leading to the deposition of amyloid plaques [304]. Although the Aβ clearance is compromised, persistent immune response induces production of proinflammatory mediators by microglial cells recruiting additional microglial cells towards plaque creating halo of activated microglial cells surrounding plaques. In addition, peripheral macrophages are also attracted towards the Aβ plaque deposition to clear Aβ peptide exacerbating neuroinflammation contributing to neurodegeneration. In AD patients, microglial cells exhibit a mixture of classical and alternate activation pathways causing irreparable damage resulting in continuous neurodegeneration.

#### 10.1.2. Astrocytes

Activated astrocytes are distributed near the vicinity of amyloid deposits in cortical pyramidal neurons in early stages of AD, which contribute to clearance of plaques by degradation and phagocytosis of accumulated Aβ in parenchyma. Like microglial cells, TLRs and RAGE pathways activate astrocytes promoting local inflammation intensifying neuronal death. Astrocyte activation causes disruption of normal activities essential for normal neuronal function leading to local neuron depolarization ultimately leading to neuronal damage. Retro-splenial cortex administration of oligomeric Aβ forms in rats revealed the presence of activated astrocyte associated with activated NF-κB, signaling molecules leading to inflammation (COX-2, TNFα and IL-1β) and expression of cell surface receptors such as SRs, proteoglycans and lipoprotein receptors, via which it binds to Aβ peptides [305,306]. Astrocytes on activation express inflammation associated factor S100β leading to dystrophic neuritis in AD patients. Activation of NF-κB regulates the secretion of chemokine and cell adhesion molecules enhancing infiltration of peripheral lymphocyte enhancing neuroinflammation ultimately leading to neurodegeneration. Astrocytes, in its activated stage, protects the brain, however, on extreme activation, it aggravates damage to the neurons hastening the progression of AD.

#### 10.1.3. Oligodendrocytes

Abnormalities in the white matter and myelin sheath have been observed in asymptomatic FAD preferentially under PS1 mutation [307]. Mutation in PS1 and Aβ accumulation alters the function and differentiation of oligodendrocyte inducing abnormal patterns in myelin basic protein (MBP), affecting homeostasis of oligodendrocytes [308]. As a result, the trophic supports provided by these cells to neurons are lost and neurons become vulnerable to oxidative stress and inflammation provoking neurodegeneration.

#### 10.1.4. Neurons

Neurons are also involved in inflammatory response which is evident by the presence of proinflammatory mediators such as COX-2-derived prostanoids, cytokines such as IL-1β and IL-18, complement and macrophage colony-stimulating factor. In addition, iNOS the inflammation induced enzyme expression in degenerating neurons is also observed in brain of AD individuals substantiating the involvement of neurons in inflammation [309]. Neurons generally produce TRM2, CD22, CD200, CD59 and fractalkine to suppress inflammation and these molecules were observed to be deficient in AD [310]. Studies on the expression of mediators of inflammation in neuronal cells are not yet completely investigated and it remains still elusive.

### 10.2. Inflammatory Mediators in AD

Aβ deposition activates the microglial cells and astrocytes to acute immune response provoking the release of mediators of inflammation-like complement factors, cytokines and chemokines (IL-1, IL-6 and TNF-α) and transforming growth factor β (TGF-β) which exhibits cascade of events with both beneficial and harmful effects.

#### 10.2.1. Complement System

The complement system regulates T-helper cell differentiation and response in adaptive immune response. In the brain, the complements are produced locally, which are dysregulated during brain trauma and neurodegenerative AD [311]. In the brain of AD patients, an enhanced level of complement components of classical pathways (C1q, C3b, C4d, C5b-9, and MAC) were observed in the vicinity of senile plaques along with microglial cells illustrating the relationship between Aβ peptide aggregation, activation of classical complement pathway and inflammatory response [312]. A recent report showed that the interaction of C1q/C3b with Aβ aggregates, and NFT activates the classical pathway, which on subsequent interaction with the C1q receptor of microglial cells, activates it leading to the clearance of Aβ and tau aggregates by phagocytosis, together with unwanted inflammation causing neurotoxicity [313]. Complement receptor 1 (CR1), widely found on the surface of phagocytic cells with binding affinity for C3b and C4b play a vital role in phagocytosis. Chibnik et al. [314] studied the genome-wide association screening (GWAS) in AD patients, where they observed an inter-relationship between CR1 gene variants with impairment in cognitive function associated with enhanced formation of amyloid plaque. Complement fragment C5a in neuronal excitotoxicity induced apoptosis, promotes chemotaxis and glial cell activation leading to the development of neurodegenerative disease. Although complement activation in AD play beneficial role in Aβ clearance, its activation becomes deregulated, promoting unwanted inflammation leading to neurotoxicity which remains unclear and need to be studied.

#### 10.2.2. Chemokines

Chemokines in the CNS were synthesized by astrocytes and microglial and its receptors are highly localized in the neurons which might be responsible for inflammation mediated neurodegeneration [315]. Expression of chemokines and their receptors promotes communication between microglia and neuronal cells leading to commencement of local inflammatory response promoting phagocytosis of Aβ peptide in early AD. This inflammation also contributes to Tau pathology accelerating progression of the disease [316]. Enhanced chemokine level recruits the phagocytic and microglial cells which co-localizes near the senile plaques during the process of chronic inflammation in AD. Enhanced level of CCL2 in CSF correlates with cognitive decline and IL-8 production by neurons is related to formation of brain-derived neurotrophic factor (BDNF) [317]. Overall, chemokines in the CNS promotes migration of local and peripheral immune cells to establish an immune response, which on chronic production, leads to inflammation mediated neurodegeneration in AD.

#### 10.2.3. Cytokines

Cytokines are non-structural soluble proteins produced by immune cells such as microglial cells and astrocytes in the CNS which play a significant role in the development of the brain during embryonic stages. An elevated level of pro-inflammatory cytokines (IL-1β, IL-6, IL-10, TNF-α, and TGF-β) was found in the CSF and brain of AD patients, illustrating the role of cytokines in aggravating AD pathology. The transgenic animal model expressing mutant hAPP protein revealed the link between the cytokines level and Aβ aggregates [318]. Aβ aggregates activate the microglial cells to produce pro-inflammatory cytokines, which in turn, activate microglial cells leading to microgliosis and astrogliosis, amplifying the cytokine level leading to neurodegeneration, amyloidosis due to upregulation of β and γ secretase affecting learning and spatial memory [319].

### 10.3. Proinflammatory Mediators

#### 10.3.1. Interleukin 1 (IL-1)

IL-1 up-regulated in early AD induces endothelial APP-β at mRNA expression which can be inter-related to enhanced Aβ, level in AD patients [320]. Microglial cells surrounding neuritic plaques (NPs) in AD patients produce IL-1, which promotes S100β synthesis in reactive astrocytes leading to dystrophic neurite formation ultimately leading to neuronal death [321]. In addition, IL-1 also promotes p38-MAP kinase activity enhancing tau hyperphosphorylation and enhances the level of neurotrophin-3 and neurogenin-1 promoting neurogenesis through promoting outgrowth. We also found that IL-1β increased mRNA and protein levels of Wnt5a, promotes neurogenesis through the Wnt5a/RhoA/ROCK/JNK pathway [322]. IL-1β is the master regulator in brain inflammatory cascade regulating the level of TNFα and IL-6. An elevated level of IL-1β was observed in the cerebral cortex and the hippocampal region of brain tissue from AD patients. IL-1β interacts with receptor (IL-1R) widely found in the dentate gyrus and pyramidal cells of hippocampal region of brain, which are highly susceptible to early AD pathogenesis early development of AD pathology [323]. IL-1β regulates the synthesis and secretion of APP in glial cells, upregulates β-secretase activity leading to increased amyloidogenic processing of APP enhancing Aβ burden, creating a vicious cycle where enhanced Aβ load, in turn, activates microglial cells leading to IL-1β production [324].

#### 10.3.2. IL-6

IL-6 is a multifaceted cytokine exhibiting dual role as anti-inflammatory myokine and proinflammatory cytokine depending on the condition, thereby, maintaining homeostasis of neuronal tissue. Serum and CSF of LOAD patients showed enhanced level of IL-6. Aβ aggregates induces the glial cells to produce IL-6 which enhances APP transcription, promotes tau hyperphosphorylation through activation of Cdk5 via cdk5/p35 pathway contributing to NFT formation serving as a bridge between AD core pathologies [325].

#### 10.3.3. Tumor Necrotic Factor Alpha (TNF-α)

TNF-α exhibits its biological activity via TNFR1 and TNFR2 receptors which was observed to be overexpressed in the hippocampal tissue and CSF of MCI and AD patients. Aβ aggregates stimulates microglial cells to produce TNF α through activation of NFκB pathway, which induces the pro-inflammatory factors involved in neuronal survival such as calbindin, Mn-SOD enzyme, BCl-2 protein and on contrary also activates glutaminase in microglial cells leading to glutamate induced excitotoxicity promoting neurodegenerative disorders [326]. TNF–α increases Aβ load by upregulating β and γ secretase activity enhancing amyloidogenic processing of APP protein. TNF–α enhances the cell adhesion molecule in vascular endothelial cells facilitating the migration of phagocytic cells and lymphocytes towards stress induced areas promoting inflammatory response [327].

#### 10.3.4. NF-κB

Transcription factor NF-κB acts as primary regulator of inflammation, which is activated in response to proinflammatory signals, TNF-α or IL-1. Aβ activates NF-κB via RAGE widely found in the glial cells and neurons in the vicinity of senile plaques enhancing the release of inflammatory markers which activates microglial cells and astrocytes provoking release of proinflammatory mediators, intensifying inflammation resulting in neurodegeneration. In addition, NF-κB also promotes TNG-α induced β secretase transcription increasing Aβ burden. Several studies revealed that utilization of NF-κB inhibitors and NSAIDs reduce NF-κB activity which lowered the Aβ1-42 level [328,329].

### 10.4. Anti-Inflammatory Mediators

#### 10.4.1. TGF-β (Tumour Growth Factor- β)

In AD patients, the level TGF-β is enhanced in the CSF, serum and brain microvascular endothelial cells which induces secretion of pro-inflammatory cytokines (IL-1β and TGF-α) [330]. TGF- β1 is the most abundant isoform of TGFβ secreted by astrocytes and its receptors widely distributed in neurons, astrocytes and microglial cells. TGF β1 primarily involves in neuroprotection by inhibiting Aβ production and deposition, regulating neuroinflammation, inhibiting GSK3β thereby attenuating tau hyperphosphorylation and enhancing the expression of antiapoptotic protein Bcl-2 and Bcl-xl [331]. TGF-β1 level is found to be decreased in plasma of AD patients. Deficiency in TGF-β1 induces impairment in TGF-β1 mediated Smad signaling pathway leading to Smad2/3 phosphorylation present in the hippocampal neurons accumulated with NFT and Aβ plaques [332].

#### 10.4.2. IL-10

Interleukin 10 (IL-10) secreted by microglial cells and astrocytes in healthy neurons in response to proinflammatory mediator inhibits cytokines such as IL-1α, IL-1β, TNF-α, IL-6 and MCP-1 to restore the brain homeostasis. IL10 level is increased in AD patients which serves as biomarker for diagnosis and progression of AD. Scientific evidence revealed that in some population, IL-10 polymorphism enhances the risk of AD [333,334].

### 10.5. Inflammatory Mediators

#### 10.5.1. Cyclooxygenases (COX)

In AD the microglial cells surrounding neuritic plaques (NPs) exhibited increased expression of COX-1 suggesting inflammation, while COX-2 expression in hippocampal CA3 region causes neurotoxicity depending on the level of NFT and Aβ and the observed cognitive impairment [335]. COX-2 increases the γ-secretase activity promoting amyloidogenic processing of APP enhancing the formation of amyloid plaque in parenchyma and prostaglandin E2 synthesis [336]. Increase in caspase-3 immunoreactivity and phosphorylation of retinoblastoma protein causing cell growth suppression was observed in triple transgenic mice model (hAPP/PS1/hCOX-2) due to increased expression of COX-2. The above fact was further substantiated by the primary cultures of cortical and hippocampal neurons derived from transgenic mice exhibited apoptotic mediated cell death [337]. Early stages of AD, Aβ aggregates promote IL-1β to enhance the COX-2 expression leading to synthesis of prostaglandin [338].

#### 10.5.2. Nitric Oxide (NO)

In a healthy state, the expression of inducible NOS (iNOS) is less, however under inflammatory conditions it is enhanced in microglia and astrocytes leading to an increased level of NO provoking oxidative stress mediated neuronal damage, synaptic dysfunction, and apoptosis of neurons [339]. Examination of neuronal tissue of AD patients illustrated that other than iNOS Aβ induced IL-1β and TNF-α also promotes NO and peroxynitrate release, which induces oxidative stress mediated mitochondrial damage and enhanced γ-secretase activity promoting Aβ formation [329,340]. Further, the NO leads to the formation NFT and thereby accelerates the pathogenesis in AD [341].

Overall, the scientific evidence reveals that inflammation exhibits significant role in the initiation and progression of AD pathogenesis. Several hypotheses revealed that production and accumulation of Aβ and inflammation converge and synergize the progression of this neurodegenerative disease. Inflammation starts very early in AD even before the formation of amyloid plaques as innate immune response to clear the Aβ fibrils, which becomes intense during the progression of disease and ends up in cell mediated immunity. In the late inflammatory response, the Aβ plaques induces microglial priming followed by recruitment of glial cells around amyloid plaques with predominant phagocytic activity for the removal of toxic Aβ fragments. Activated astroglial cells, together with peripheral monocytes, invade CNS forming secondary cellular corolla surrounding amyloid plaques. These immune cells produce bulk cytokines and chemokines which along with immune-related molecules such as antibodies, complement, complement-related proteins, MHC proteins, and inflammasome protein complexes activates late inflammatory response of late AD pathology. TREM2-dependent activation of microglia with disease-mitigating properties supports the fact that late AD inflammation represents a tissue-resolution stage. In the end stages of AD, CNS inflammation becomes less relevant as it declines with senescence. Despite much evidence, there remains a knowledge gap on the cells associated with AD and the pathway which link Aβ accumulation and on-going inflammation. Unravelling these mechanisms will help in identifying new therapeutic molecules in combating AD.

**Table 1:** Receptors associated with inflammatory response in AD.

Model System	CNS Cells Expressing Receptor	Ligands	Role in AD Pathogenesis	References
Complement receptors (CR1, CR3) CD88	Neurons, microglial cells, astrocytes, and oligodendrocyte	C3b, C4b C3 C5a	Neuroinflammation, uptake and clearance of Aβ	[342,343,344]
FPRL1 and FPRL2	Macrophages, glial cells, astrocytes	Aβ	Proinflammation, Aβ_42_ internalization, formation of fibrillar aggregates, phagocyte chemotaxis and oxidant stress	[251,298,345,346]
Scavenger receptor (SR-A) CD36 (SR-B) LDLR, RAGE, LRP1	Microglia, human monocytes astrocytes Macrophages neurons	Aβ, β-sheet fibrils, HMGB1	Aβ clearance, synaptic dysfunction, neuroinflammation, production of chemokines, and neurotoxic ROS, NLRP3 activation	[248,249,250,287,288,302,347,348,349,350,351]
Toll-like receptors (TLR2 TLR4, TLR2, TLR9)	Microglia, astrocytes	LPS, Aβ	LTP deficit and neuronal death, neuroinflammation, Aβ uptake and clearance.	[288,289,352,353,354]
CX3CR1	Microglia, neurons, astrocytes	CX3CL	Neuroprotection against AD. AD patients showed reduced level of CX3CR1 which led to enhanced activation of microglial cells with enhanced tau phosphorylation	[355]
TREM2	Microglia and neurons	Aβ	Microglial depolarization, apoptosis activation of Wnt/β-catenin leads to inflammation.	[356,357]
CD33	Microglia	Aβ	Increased expression of CD33 attenuates Aβ uptake leading facilitating plaque formation	[358]
NALP3/NLRP3	Microglia, macrophage cells	Aβ and other mediators	Enhanced caspase-1 activity leading to IL-1β and IL-18 mediated neuroinflammation	[300,301,302]

## 11. Conclusions

To start with review elaborated in detail, the genomics and proteomics modifications of APP and secretases mediated amyloidogenic processing of APP in both cell membrane and cellular organelles leading to the release of insoluble Aβ peptides which tends to aggregate as oligomers and plaques in synaptic junction causing organelle dysfunction and disease progression. Despite of multiple etiological factors, mounting evidence hypothesised that Aβ is the key triggering factor inducing AD pathogenesis via hyperphosphorylation of tau protein, ER stress, Golgi stress/fragmentation, mitochondrial dysfunction, lysosome dysfunction, inflammation, obstruction of the synaptic communication and genomic dysregulation. Current symptomatic treatment approved by FDA for the AD therapeutics involves AChE inhibitors and NMDA receptor antagonist which can only slow down the progression of disease. As AD is a complex disorder involving several biochemical pathways, drugs with multipotent targeting ability are needed for the AD therapy. Several treatment strategies have been proposed which showed positive results in preclinical trials but suffered limitation under human trial due to blood brain barrier reducing the bioavailability of the drug to brain. Hence, an effective treatment strategy for the prevention and cure of AD is still at the developmental stage.

Recent evidence suggests that nutritional supplementation rich in antioxidants, vitamin B12 and folic acid attenuates the fibrillation of Aβ α-synuclein and p-tau, consequently, inhibits Aβ mediated toxicity and attenuates the neuronal inflammation [359,360,361]. Hence combinatorial therapy of a nutritional diet along with less toxic natural drugs inhibiting Aβ production will be effective for AD therapy. Drugs screened for AD therapy should abide the following properties (i) targeting the amyloidogenic pathway proteins either in genomic/proteomic level, (ii) potentially activating the enzymes, or directly modifying the Aβ hydrophobic properties upon binding, and (iii) developing epitope-specific monoclonal antibodies. However, most of the pharmaceutical industries approaching in the above aspects, observed that the drugs screened were toxic in nature and less efficient due to poor bioavailability. Few drugs have entered successfully into Phase3 clinical trials [362]. Complete understanding of disease pathogenesis, pharmacokinetics and bioavailability of drug is necessary to solve the puzzle in AD therapy. As this review unwinds all the plausible mechanisms leading to AD pathogenesis, it provides new insightsinto identifying the key targets for the treatment of AD.

## Data Availability

Not applicable.
